# Supplementary material for: Simultaneous cellular and molecular phenotyping of embryonic mutants using single-cell regulatory trajectories
Source: Dev Cell. 2022 Feb 28;57(4):496–511.e8. doi: 10.1016/j.devcel.2022.01.016 (PMC8893321; doi:10.1016/j.devcel.2022.01.016)
Supplement: Document S1. Figures S1–S6 [file mmc1.pdf]

Developmental Cell, Volume *57*

Supplemental information

Simultaneous cellular and molecular phenotyping of embryonic mutants using single-cell regulatory trajectories

Stefano Secchia, Mattia Forneris, Tobias Heinen, Oliver Stegle, and Eileen E.M. Furlong

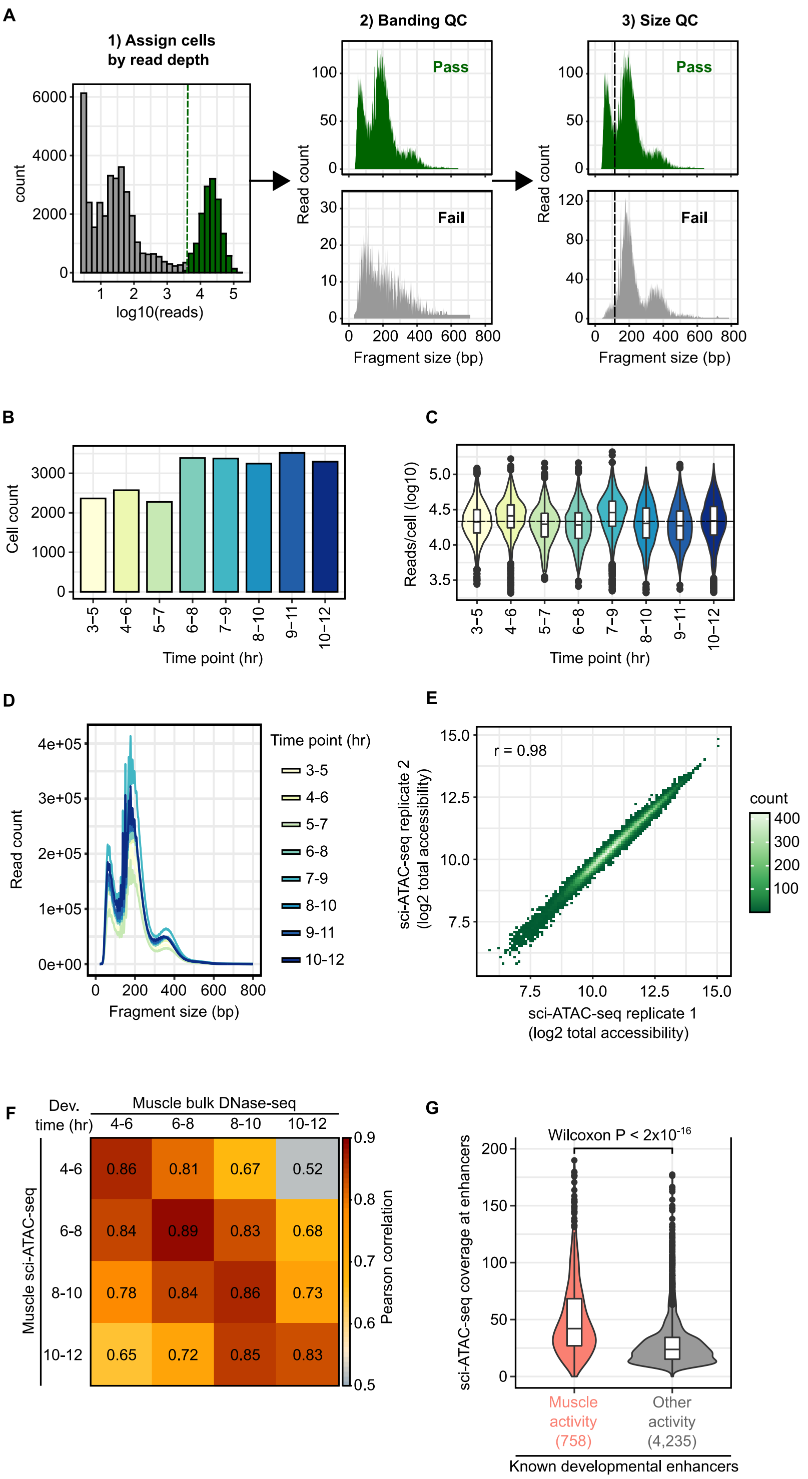

Figure S1 (related to Figure 1): sci-ATAC-seq during a dense time course of mesoderm development

(A) Quality control of sci-ATAC-seq libraries using a three-step filtering procedure. Only cells passing an initial read depth filter (step 1), with a clear nucleosome banding pattern (step 2) and fragment size distribution (step 3) were retained for downstream analysis. **(B)** Number of cells profiled per time point for the wild-type mesoderm/muscle time course. **(C)** Coverage (log10 unique reads) per cell per time point for the wild-type mesoderm/muscle time course. Dashed line indicates the median coverage per cell across the entire dataset (21,649 reads/cell). **(D)** Fragment size distribution per time point displays the expected nucleosome banding pattern for ATAC-seq libraries. **(E)** Correlation of sci-ATAC-seq replicates (batch 1 and 2) in accessibility across 42,076 merged ATAC-seq peaks called on each time point. r indicates the Pearson's correlation coefficient, showing little batch effect. **(F)** Correlation between bulk DNase-seq accessibility profiles from FAC sorted mesoderm/muscle cells (Reddington et al., 2020) and aggregated pseudobulk sci-ATAC-seq profiles for time-matched samples. **(G)** sci-ATAC-seq coverage at characterized mesoderm/muscle enhancers (red) and enhancers active in other embryonic tissues (non-muscle, grey).

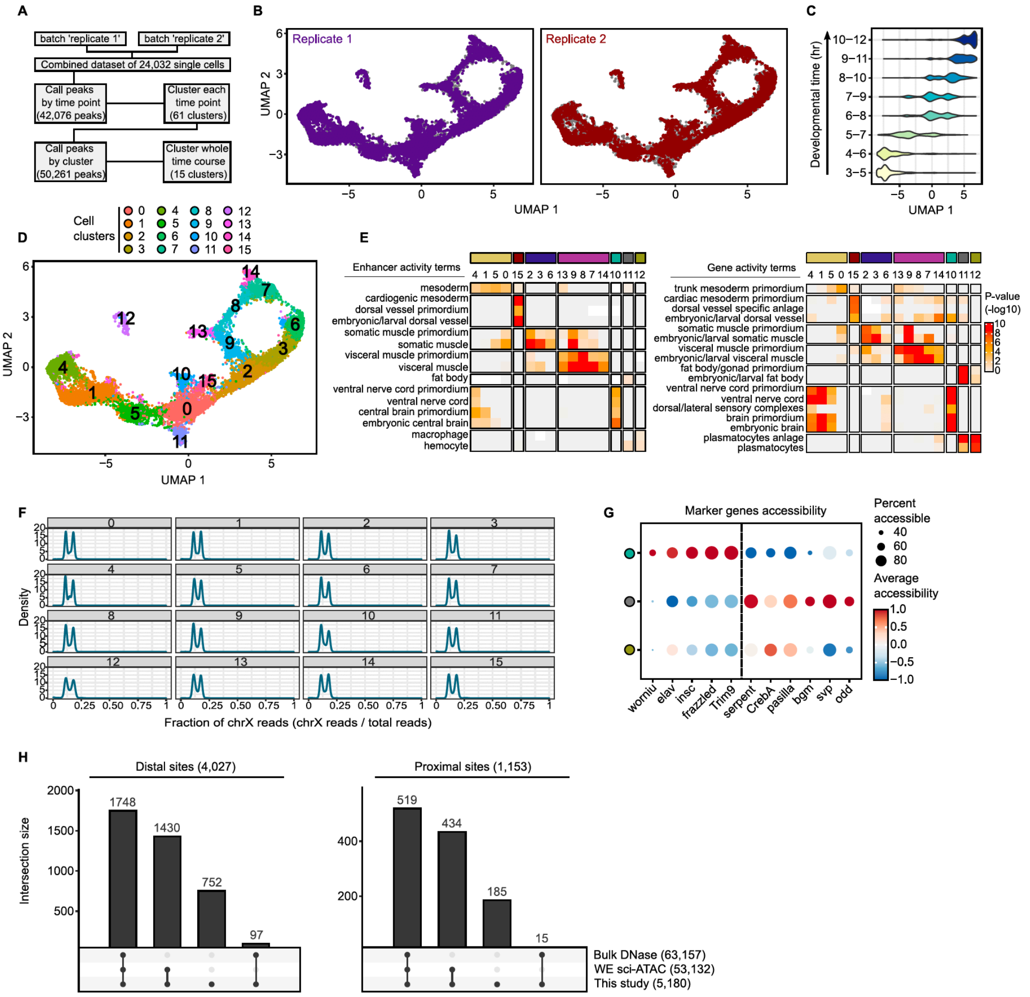

Figure S2 (related to Figures 1-2): Constructing, annotating and analyzing an atlas of chromatin accessibility during a time-course of mesoderm/muscle development

(A) Overview of data processing and clustering strategy. After cell filtering (Fig. S1A), the two sci-ATAC-seq batches were merged giving a combined set of 24,032 mesodermal (Mef2+) cells. To maximise sensitivity to detect peaks at each stage and cell type, peaks were first called separately for each timepoint (yielding a combined set of 42,076 peaks), which were used to cluster each time point individually (using Seurat v3.2.2), yielding 61 cell clusters. Accessibility peaks were identified again in each cell cluster, identifying 50,261 merged peaks used for clustering the whole wild-type time-course. **(B)** UMAP visualization of the whole mesoderm/muscle time course, highlighting cells belonging to each batch in purple (rep 1) or red (rep 2). Cells from each batch are evenly distributed throughout the time-course, suggesting little or no batch effect, keeping with the high correlation between batches (Fig. S1E). **(C)** Cell distribution over the first UMAP dimension per time point, showing the gradual progression along developmental time. **(D)** UMAP visualization of the full mesoderm/muscle time course with cells coloured by their respective Seurat defined cluster. **(E)** Enrichment for enhancers with tissue activity (left) and genes with tissue expression (right) terms in each cluster. Fisher's exact test P-value (-log10) displayed as a heatmap. Cell clusters are grouped by the inferred cell type annotations (top bars), colours correspond to Figure 1D. **(F)** As embryo collections are a mix of male and females, we assessed if there are sex-dependent effects. Each nucleus was molecularly sexed using the fraction of reads mapping to the X Chr, as female nuclei have roughly double the number of X-reads than males. Density plots show the fraction of reads from the X Chr (x-axis) for nuclei in each cluster (corresponding cluster number from (D) indicated on top). Each cluster has a bimodal distribution among nuclei in the ratio of X-Chr reads, indicating little or no sex-biases in each muscle cluster. **(G)** Marker genes accessibility for non-myogenic cell populations (top to bottom: neuro, fat body, hemocytes). The colour scale indicates gene average accessibility (Z-score), while dot size indicates the percentage of cells in which the gene is accessible. Annotation colours correspond to Figure 1D. **(H)** Upset plot showing the intersection between differentially accessible (DA) ATAC-seq peaks discovered here (5,180 peaks from Fig. 2D) compared to 63,157 peaks from tissue-specific bulk DNase-seq (Reddington et al., 2020) and 53,132 peaks from whole-embryo (WE) shot-gun sci-ATAC-seq (Cusanovich et al., 2018a). The DA sites are divided into distal (left, >500bp from a Transcriptional Start Site (TSS)) and TSS-proximal sites (right). An intersection is counted if at least 25% of a peak in this study overlaps a peak in another dataset.

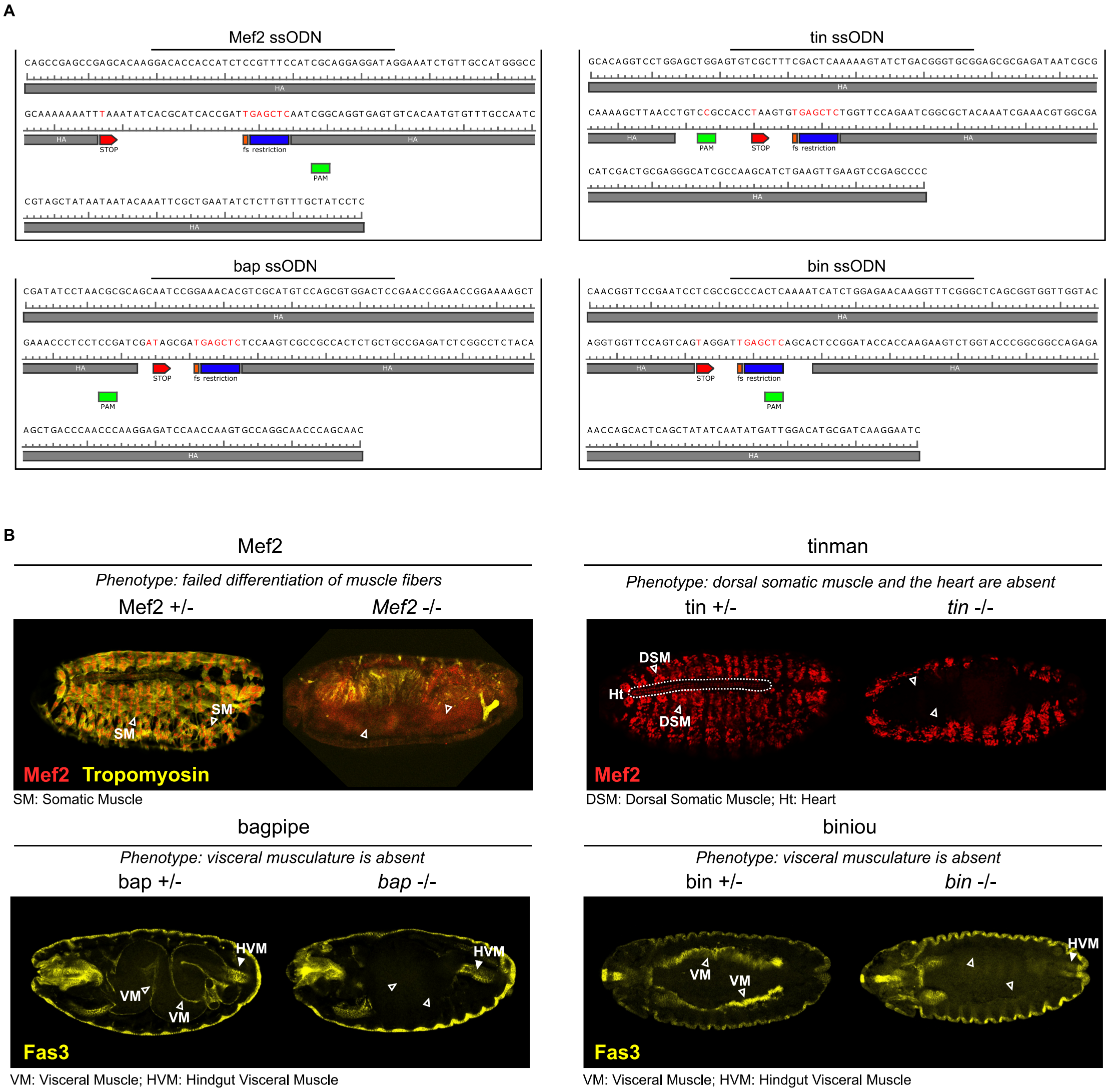

Figure S3 (related to Figures 4-5): Generation and validation of new loss-of-function mutants for four mesodermal transcription factors

(A) Design of the single stranded oligonucleotides (ssODNs) for the *Mef2*, *tin* (*tinman*), *bap* (*bagpipe*), and *bin* (*biniou*) loci, which were used as homology directed repair (HDR) templates following a Cas9 induced double strand break. Nucleotides that differ from the isogenic vasa-Cas9 locus are indicated in red. For *Mef2*, *tin* and bin, the introduced mutation recapitulates the characterised loss-of-function allele, but is now generated in a clean isogenic background common to all four mutants. This previously characterized single point nonsense mutation (indicated by the red "STOP" box), in addition to a restriction site for SacI (GAGCTC; blue box) and a thymine nucleotide causing a nonsense frame-shift mutation (orange box) were introduced in each locus. Additional single point mutations were introduced in either the PAM site or the gRNA seed to prevent re-cutting by Cas9. **(B)** All four CRISPR generated new alleles recapitulate the characterised loss-of-function phenotypes for each gene. Upper left panels: Double immunostaining of Mef2 (red) and Tropomyosin (yellow) in heterozygous (+/-; identified by LacZ expression on the balancer chromosome) and homozygous (-/-) *Mef2* mutant stage 16 embryos. While the *Mef2* +/- embryo has stereotypically patterned somatic muscle (SM), the *Mef2* -/- embryo has an absence of Mef2 protein and little or no differentiated somatic muscle. Upper right panels: Immunostaining of Mef2 (red) in *tin* heterozygous (+/-) and homozygous (-/-) mutant stage 16 embryos. The dorsal somatic muscle (DSM) and heart muscle (Ht) indicated in *tin* +/- embryos are missing in the *tin* -/- embryo, in addition to the visceral muscle (not shown). Lower panels: Immunostaining of Fas3 (yellow) as a marker for visceral muscle (VM) in *bap* (left) and *bin* (right) heterozygous (+/-) and homozygous (-/-) mutant embryos. Note the complete absence of circular trunk visceral muscle in both mutants, while the hindgut VM (HVM) is unaffected. All embryos are orientated anterior to the left, and dorsal up.

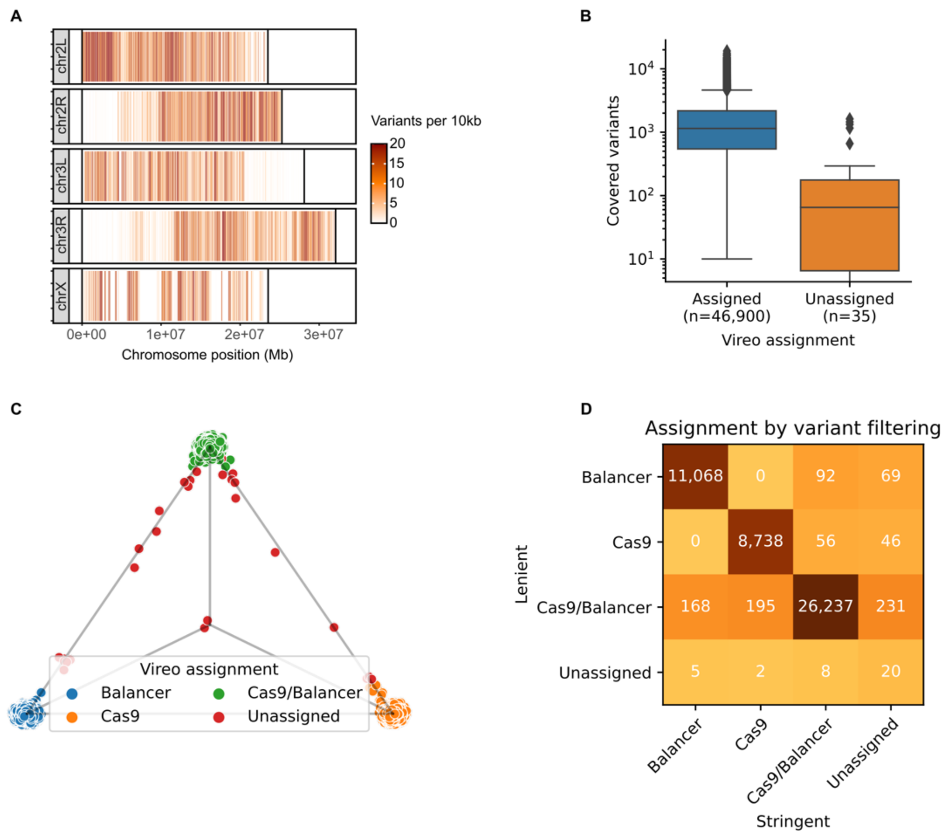

Figure S4 (related to Figures 4-5): Digital nuclear genotyping

(A) The density of genetic variant between the Cas9 isogenic strain used to make the loss-of-function mutants (sequenced in this study) and the balancer chromosome (sequenced in our previous study (Ghavi-Helm et al, 2019) across chromosomes 2, 3 and X. **(B)** Box plots showing the total number of SNP variants per nuclei with a genotype assigned (blue) or unassigned (orange) by Vireo (Huang et al., 2019). **(C)** Triangle plot showing assignment probabilities for each genotype, which are represented as points in the triangle. The center of the triangle corresponds to the vector (1/3, 1/3, 1/3), while the vertices represent a probability of 1 for Balancer (bottom left), Cas9 (bottom right) and Cas9/Balancer (top), respectively. Posterior probabilities are focused near the vertices of the triangle, indicating low posterior uncertainty. **(D)** Confusion matrix for the genotype assignment using variants called with a stringent or a lenient filter, demonstrating that the model is robust to different reference annotations.

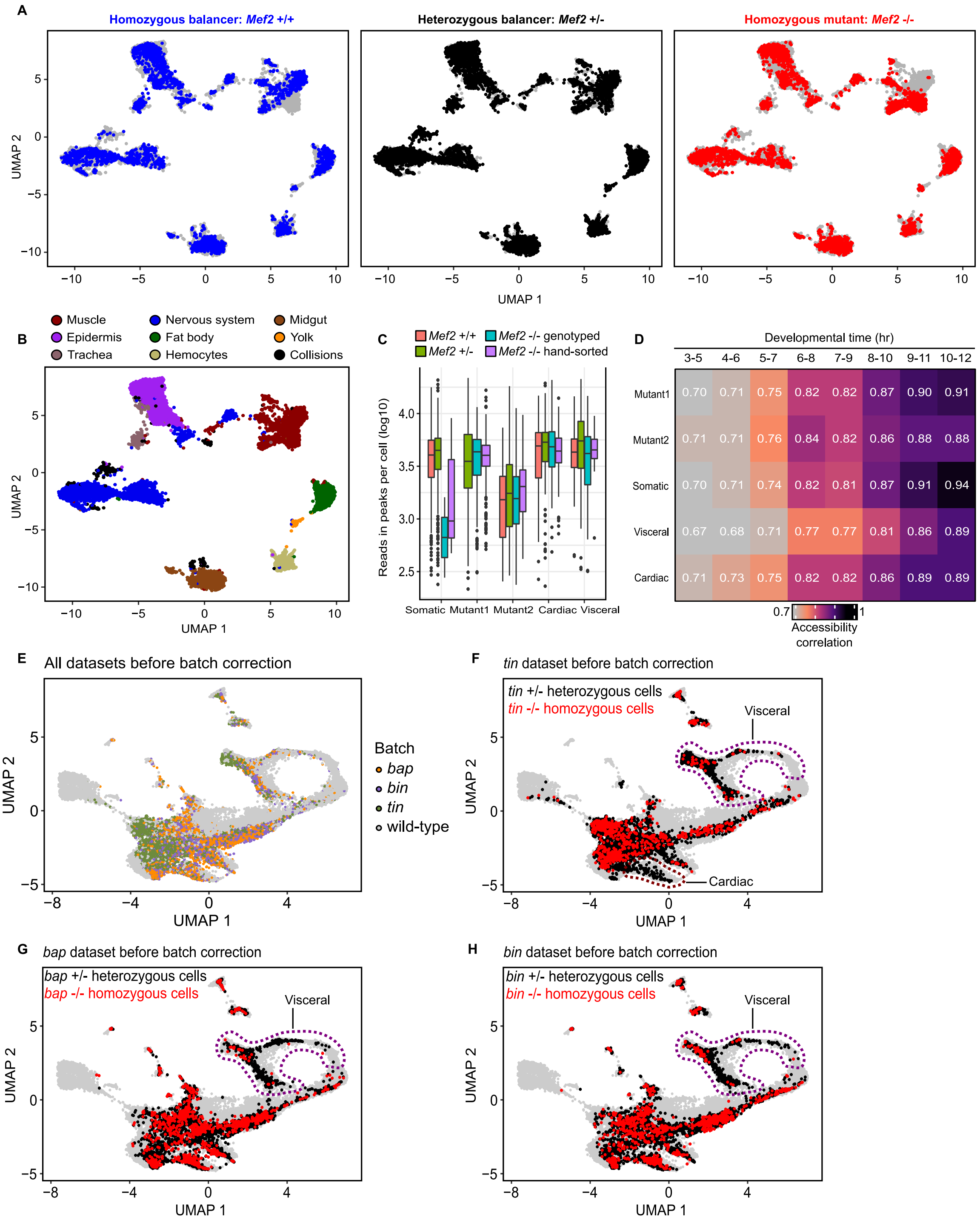

Figure S5 (related to Figures 4-5): Clustering, annotation and analysis of *Mef2*, *tin*, *bap*, *bin* mutants
(A) UMAP visualization of whole-embryo cell clustering from the *Mef2* sample; homozygous balancer/balancer (left, blue), heterozygous Mef2^{lof}/balancer (+/-, middle, black), homozygous Mef2^{lof} (-/-, right, red) cells are highlighted. Cell clustering is not obviously impacted by genotype, indicating little batch effect and therefore no need for correction. **(B)** Same as (A), cells coloured by inferred cell type, revealing 8 populations, including muscle cells (red). **(C)** Reads in peaks per cell (log10 scale) per cell cluster from Fig. 4d, split by genotype. Mutant 2 cluster has lower read counts/cell. **(D)** Pearson correlation of open chromatin for clusters in Fig. 4d in time point aggregate samples from the wild-type time course. Mutant 1 cells have the highest correlation to cells at 10-12hr, matching the specific time point of their sampling (10-12 hr). **(E)** UMAP visualization before Harmony batch correction of wild-type sci-ATAC-seq time course re-clustered with single cell datasets from *tinman* (*tin*), *bagpipe* (*bap*) and *biniou* (*bin*). All cells from each dataset (batch) are highlighted (*bap* - orange, *bin* - purple, *tin* - green, wild-type - grey). **(F-H)** Same as (E), but for each dataset (batch) the heterozygous (+/-; black) and homozygous mutant (-\-; red) cells are highlighted. The cardiac and visceral muscle populations are highlighted with dashed lines.

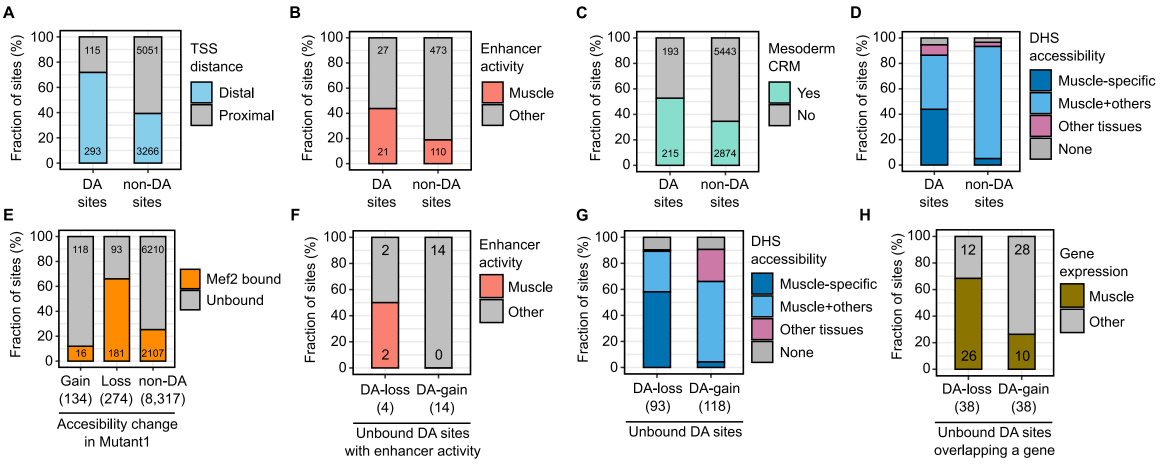

Figure S6 (related to Figure 6): Characterization of regulatory changes in *Mef2* mutants

(A) Fraction of differentially accessible (DA) or non-DA sites in Mutant 1 cells that are gene distal (light blue) or gene proximal (grey). Gene distal is defined as >500bp from an annotated transcriptional start site. **(B)** Fraction of *Mef2* -/- DA or non-DA sites that overlap characterized embryonic enhancers with demonstrated activity in muscle (red) or other tissues (grey). **(C)** Fraction of *Mef2* -/- DA or non-DA sites overlapping mesoderm *cis*-regulatory modules (CRMs), defined by ChIP against 5 mesoderm/muscle transcription factors (Zinzen et al., 2009). **(D)** Fraction of DA or non-DA sites overlapping DNase I Hypersensitive Sites (DHSs) in muscle only (Muscle-specific), in muscle and other tissues (Muscle+others), in non-muscle tissues (Other tissues) or that do not overlap a DHS (None). DHS data from Reddington et al, 2020. **(E)** Fraction of Mef2-bound (orange) and unbound (grey) sites, split by chromatin accessibility change in Mutant1 cells, either gain or loss of accessibility, or unchanged (non-DA) sites. **(F)** Fraction of Mef2-unbound DA sites that lose (DA-loss) or gain (DA-gain) accessibility in Mutant1 overlapping characterised enhancers active in muscle (red) or other tissues (grey). **(G)** Fraction of Mef2-unbound DA sites that lose (DA-loss) or gain (DA-gain) accessibility in Mutant1 that overlap DHS in muscle only (Muscle-specific), in muscle and other tissues (Muscle+others), in non-muscle tissues (Other tissues) or that do not overlap a DHS (None). DHS data from Reddington et al, 2020. **(H)** Fraction of Mef2-unbound DA sites that lose (DA-loss) or gain (DA-gain) accessibility in Mutant1 overlapping genes with characterized expression in muscle (olive) or non-muscle tissues (other, grey). Gene expression data from the BDGP database.
