# Supplementary material for: Simultaneous cellular and molecular phenotyping of embryonic mutants using single-cell regulatory trajectories
Source: Dev Cell. 2022 Feb 28;57(4):496–511.e8. doi: 10.1016/j.devcel.2022.01.016 (PMC8893321; doi:10.1016/j.devcel.2022.01.016)
Supplement: Document S2. Article plus supplemental information [file mmc8.pdf]

Developmental Cell

Article

Simultaneous cellular and molecular phenotyping of embryonic mutants using single-cell regulatory trajectories

Graphical abstract

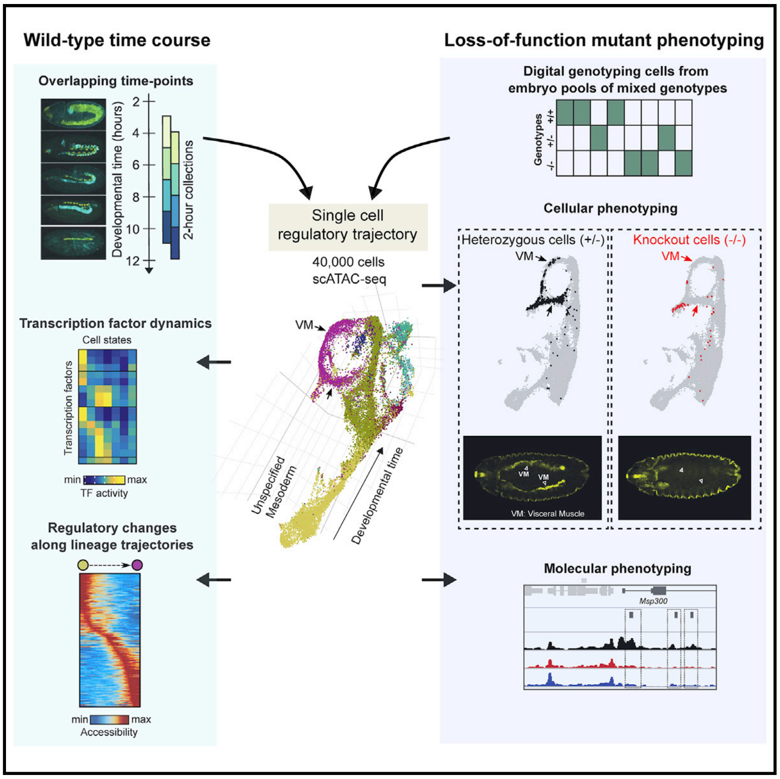

Highlights

- scATAC time course constructs regulatory trajectories of *Drosophila* muscle lineages
- Combining a wild-type trajectory with mutants identifies and quantifies phenotypes *de novo*
- Digital nuclear genotyping enables the processing of pooled embryos of mixed genotypes
- This framework simultaneously uncovers cellular and molecular phenotypes in embryos

Authors

Stefano Secchia, Mattia Forneris, Tobias Heinen, Oliver Stegle, Eileen E.M. Furlong

Correspondence

furlong@embl.de

In brief

By applying scATAC-seq over a dense time course of mesoderm development, Secchia et al. generate regulatory trajectories of multiple muscle lineages in *Drosophila*. They demonstrate that integrating this with loss-of-function transcription-factor mutants is sufficient to uncover cellular phenotypes *de novo*, while simultaneously providing new insights into the factor's molecular function.

Secchia et al., 2022, Developmental Cell *57*, 496–511
February 28, 2022 © 2022 The Author(s). Published by Elsevier Inc.
https://doi.org/10.1016/j.devcel.2022.01.016

CellPress
OPEN ACCESS

Developmental Cell

Article

Simultaneous cellular and molecular phenotyping of embryonic mutants using single-cell regulatory trajectories

Stefano Secchia,[1,2] Mattia Forneris,[1] Tobias Heinen,[3,4] Oliver Stegle,[1,3] and Eileen E.M. Furlong[1,5,*]
[1]European Molecular Biology Laboratory (EMBL), Genome Biology Unit, 69117 Heidelberg, Baden-Württemberg, Germany
[2]Collaboration for Joint PhD Degree between EMBL and Heidelberg University, Faculty of Biosciences, Baden-Württemberg, Germany
[3]Division of Computational Genomics and Systems Genetics, German Cancer Research Center (DKFZ), 69120 Heidelberg, Baden-Württemberg, Germany
[4]Heidelberg University, Faculty of Mathematics and Computer Science, 69120 Heidelberg, Baden-Württemberg, Germany
[5]Lead contact
*Correspondence: furlong@embl.de
https://doi.org/10.1016/j.devcel.2022.01.016

SUMMARY

Developmental progression and cellular diversity are largely driven by transcription factors (TFs); yet, characterizing their loss-of-function phenotypes remains challenging and often disconnected from their underlying molecular mechanisms. Here, we combine single-cell regulatory genomics with loss-of-function mutants to jointly assess both cellular and molecular phenotypes. Performing sci-ATAC-seq at eight overlapping time points during *Drosophila* mesoderm development could reconstruct the developmental trajectories of all major muscle types and reveal the TFs and enhancers involved. To systematically assess mutant phenotypes, we developed a single-nucleus genotyping strategy to process embryo pools of mixed genotypes. Applying this to four TF mutants could identify and quantify their characterized phenotypes *de novo* and discover new ones, while simultaneously revealing their regulatory input and mode of action. Our approach is a general framework to dissect the functional input of TFs in a systematic, unbiased manner, identifying both cellular and molecular phenotypes at a scale and resolution that has not been feasible before.

INTRODUCTION

Understanding the progression and regulation of cell lineages is a major goal of developmental biology. Genetic studies traditionally addressed this either at the cellular level, describing high-level tissue abnormalities by immunostaining mutant embryos, or at a molecular level using genomics or biochemical approaches, with limited integration between the two. The development of single-cell methods provides new opportunities to change this, having the potential to obtain a more fine-grained description of mutant phenotypes at both cellular and molecular levels. Recent studies have demonstrated the power of single-cell transcriptomics to uncover cellular diversity, identify new cell states, and chart cellular trajectories during embryonic development. For example, single-cell RNA-seq (scRNA-seq) can map the development of tissues—e.g., the *Drosophila* optic lobe (Özel et al., 2021), aging brain (Davie et al., 2018), and mouse heart (Tyser et al., 2021)—and whole organisms, as shown for mice (Argelaguet et al., 2019; Pijuan-Sala et al., 2019), zebrafish (Farrell et al., 2018; Wagner et al., 2018), *Xenopus* (Briggs et al., 2018), and planarians (Plass et al., 2018).

Expression states are regulated by transcription factors (TFs), which drive both the diversity and progression of cell lineages. Many TFs act through enhancers to regulate a specific pattern of gene expression. TFs thereby specify and maintain cellular identity, giving a cell or tissue much of its morphological and functional characteristics (Farley et al., 2015; Furlong and Levine, 2018; Reiter et al., 2017; Spitz and Furlong, 2012). Deciphering how developmental lineages are regulated therefore requires an understanding of both the TFs involved and the enhancers they regulate. While scRNA-seq provides information on the former, the function and direct contribution of these factors need to be inferred. Single-cell regulatory genomics methods, such as single-cell ATAC-seq (sci-ATAC-seq) (Minnoye et al., 2021), provide a direct approach to uncover enhancer usage in different tissues and stages of development, as recently demonstrated during *Drosophila* (Cusanovich et al., 2018a), mouse (Argelaguet et al., 2019; Pijuan-Sala et al., 2020), and human (Domcke et al., 2020) embryogenesis. Combining such information, obtained through a high-resolution time course of embryogenesis, with loss-of-function mutants holds the promise of uncovering the functional role of developmental factors at both a cellular and molecular level.

To explore this, we selected mesoderm specification into different muscle primordia as a well-studied model system. This germ layer gives rise to all major muscle types from flies

This is an open access article under the CC BY license (http://creativecommons.org/licenses/by/4.0/).

CellPress
OPEN ACCESS

to humans, and the key TFs regulating the subdivision of the mesoderm into different muscle lineages are known and highly conserved. Seminal genetic screens in *Drosophila* uncovered the functional requirement of many of these factors, describing high-level phenotypes such as missing or abnormal muscles (Azpiazu and Frasch, 1993; Bour et al., 1995; Lilly et al., 1995; Zaffran et al., 2001). There are also extensive molecular data describing TF binding and enhancer usage during wild-type mesoderm-to-muscle development during *Drosophila* embryogenesis (Ciglar et al., 2014; Jakobsen et al., 2007; Junion et al., 2012; Liu et al., 2009; Reddington et al., 2020; Sandmann et al., 2006, 2007a; Zinzen et al., 2009).

To better integrate these two, i.e., high-level tissue phenotypes with the molecular input of their developmental regulators, we first generated a dense time course of single-cell regulatory changes during mesoderm development in wild-type *Drosophila* embryos. Performing sci-ATAC-seq across eight overlapping embryonic time points captured a continuum of regulatory transitions as cells move from multipotency down different developmental lineages. We then use these regulatory trajectories to examine the phenotypes of four essential TFs in loss-of-function mutants, providing a high-resolution view of their functional impact at both a cellular and molecular level. We demonstrate that this approach can pinpoint and quantify known tissue defects *de novo* and uncover new more subtle phenotypes, while simultaneously obtaining a deeper understanding of the molecular features and regulatory role of the TFs' input. Taken together, this study provides a framework to systematically assess the role of developmental regulators at multiple levels and serves as a forerunner for building predictive networks of tissue and organismal development.

RESULTS

A single-cell atlas of chromatin accessibility during a comprehensive time course of mesoderm development

To capture the regulatory trajectories of different muscle lineages, we profiled sci-ATAC-seq in eight overlapping, rather than adjacent, 2 h tightly staged embryo collections to resolve continuous single-cell trajectories (Figure 1A). This time course initiates shortly after gastrulation when cells are multipotent (3–5 h) and continues through the stages of lineage commitment to terminal differentiation (10–12 h), ensuring that all major developmental transitions are captured. The nuclei in each time point were obtained from hundreds of embryos and naturally sample an even finer range of developmental states. For each 2-h staged collection, embryos were formaldehyde fixed and intact nuclei were isolated and stained with a mesodermal/muscle marker (myocyte enhancer factor 2 [Mef2]) and FAC sorted to >95% purity (Figure 1A) using our optimized batch isolation of tissue-specific (BiTS) nuclei protocol (Reddington et al., 2020).

We performed two sci-ATAC-seq "replicates" per time point, consisting of independent embryo collections and mesodermal nuclei sorting, which resulted in a combined dataset of 24,032 single mesodermal nuclei (subsequently referred to as cells) that passed quality filters (STAR Methods; Figure S1A; Table S1). Through protocol optimizations, we roughly doubled the read coverage per cell compared with our previous study (Cusanovich et al., 2018a), obtaining a median of 21,649 unique reads/cell. This is comparable with sci-ATAC-seq studies in mice (Cusanovich et al., 2018b), but as the *Drosophila* genome is ~25-fold smaller, this yields a comprehensive coverage of occupied enhancers in each individual cell. The total number of cells, and median coverage per cell, was generally consistent across time (Figures S1B and S1C). Four lines of evidence attest to the high quality of these sci-ATAC-seq data: (1) the insert size has the expected nucleosomal distribution (Figure S1D); (2) pseudobulk profiles, created by aggregating single cells, are highly correlated among "replicate" batches (Figure S1E); and (3) importantly our single-cell mesodermal data are highly correlated with bulk DNase-seq profiles from FAC-purified muscle populations (Figure S1F); and (4) they overlap characterized mesoderm/muscle enhancers (Figures 1B and S1G).

To maximize sensitivity to detect open chromatin regions over the entire time course, we performed a first round of clustering of each time point separately (yielding 61 clusters in total) and called peaks for each cell cluster, generating a merged set of 50,261 unique peaks (STAR Methods; Figure S2A). Cell clustering was performed on the resulting cell-by-peak accessibility matrix using cisTopic (Bravo González-Blas et al., 2019), which outperforms other methods on continuous populations (Chen et al., 2019). Unsupervised clustering of all cells from all time points revealed 15 clusters organized in a tree-like structure that naturally reflects the temporal order of the embryonic collections (Figures 1C, S2B, and S2C). Cells from the early time points are relatively uniform in their regulatory landscape and form a trunk that represents nascent unspecified mesoderm. The accessibility landscape markedly diversifies at ~6–8 h of development, causing cells to branch along different trajectories (Figure 1C). Although the clustering algorithm had no prior information, this matches the time window uncovered by genetic studies in the 1990's for the subdivision of the mesoderm into different muscle primordia (Azpiazu and Frasch, 1993; Azpiazu et al., 1996; Riechmann et al., 1997).

To determine the identity of the 15 major cell clusters (Figure S2D), we assessed the overrepresentation of tissue terms using two extensive resources: (1) curated enhancers with embryonic activity *in vivo* (Bonn et al., 2012a; Kvon et al., 2014; Rivera et al., 2019) and (2) gene expression throughout embryogenesis (Tomancak et al., 2002) (Figure S2E). These independent approaches gave highly concordant annotations and resolved the early mesoderm population and the three major myogenic lineages: the somatic, visceral, and cardiac muscles (Figure 1D), indicating that they have distinct chromatin accessibility landscapes and require differential usage of regulatory elements. As specification begins halfway through our time course, unspecified mesodermal cells constitute the largest population of cells, while the cardiogenic mesoderm and resulting heart muscle is the least abundant (Figure 1D). The fact that we can detect cardiomyocytes, a rare cell population consisting of only 104 cells per embryo (Reim and Frasch, 2010), indicates that we have comprehensively sampled the diversity of myogenic cell types at these stages. The continuum of muscle development is reflected in the distribution of different cell populations, which shifts over time with the early uncommitted progenitors being gradually replaced by the terminal muscle types (Figure 1E). We also identified three small nonmyogenic populations. Fat body and hemocytes are both derived from the mesoderm and accordingly are

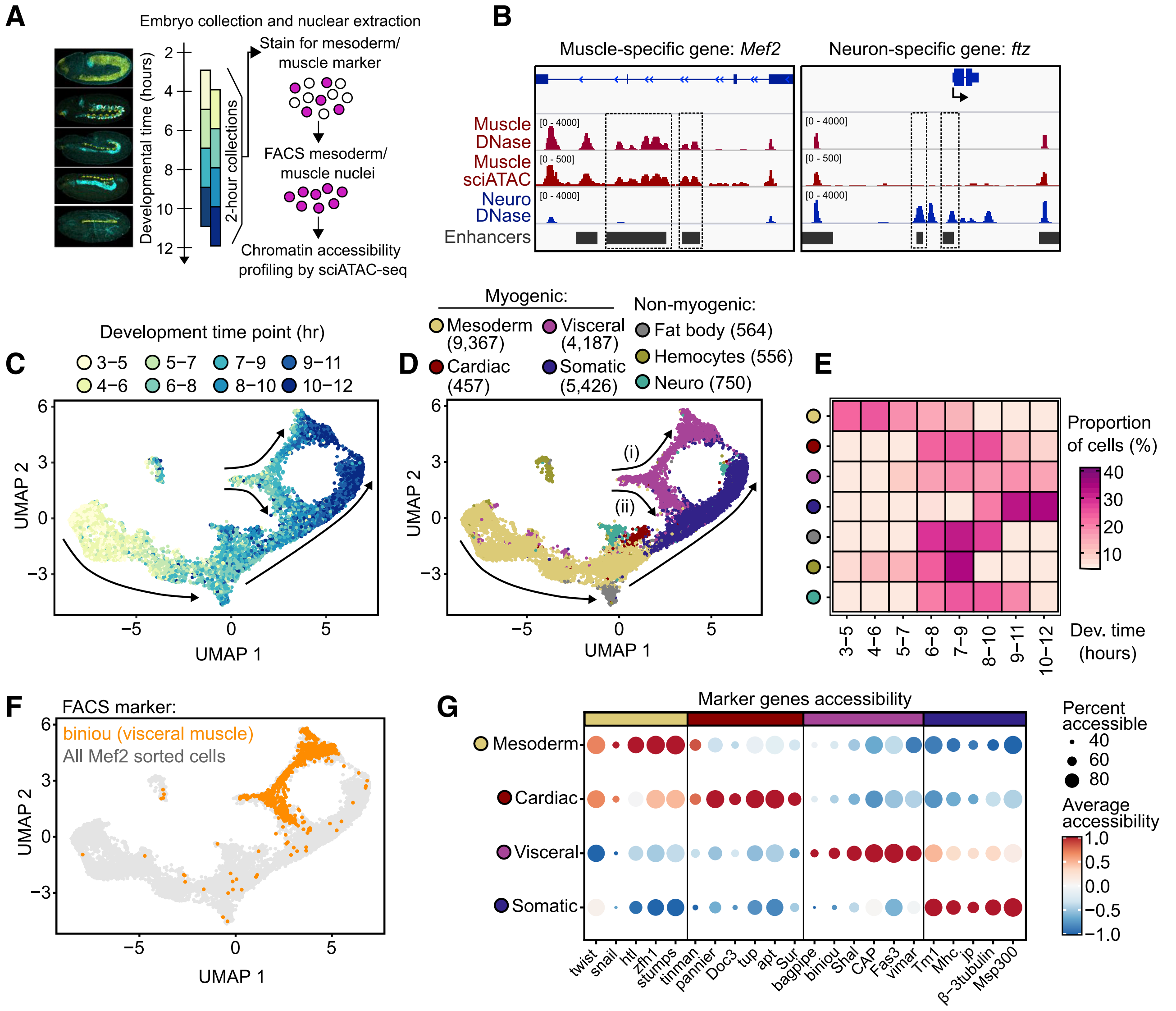

Figure 1. A single-cell regulatory time course of mesoderm specification to different muscle lineages

(A) Staged embryos were collected from 8 overlapping 2-h windows and mesoderm/muscle nuclei FAC sorted based on Mef2 expression prior to sci-ATAC-seq. Tinman (yellow) and Biniou (green) immunostainings are shown (left).

(B) Bulk DNase-seq and aggregated sci-ATAC-seq chromatin accessibility at a muscle (left, *Mef2*) and neuron (right, *ftz*) specific gene. Characterized enhancers indicated in gray.

(C) UMAP visualization of all nuclei over the time course, colored by the time point of the embryo collection.

(D) Same as (C), colored by cell-type annotation.

(E) Percentage of cells in each time point (hours) by cell population.

(F) Same as (C), gray cells were FAC sorted using a general mesoderm/muscle marker (Mef2), orange cells using a visceral-muscle-specific marker (Biniou).

(G) Dotplot of marker-gene accessibility in each muscle type. Color scale indicates gene average accessibility (Z score); dot size, the percentage of cells in which the gene is accessible. Nonmyogenic populations are shown in Figure S2G.

present at the specification stages but absent at later time points (Figure 1E). The third nonmyogenic population are neural cells, which may come from a subpopulation of Mef2-expressing cells within the mushroom body of the brain (Crittenden et al., 2018). We observed no sex biases between different muscle clusters (Figure S2F).

To validate the cell-type assignment, we first FAC sorted cells from the visceral muscle using the lineage-specific marker Biniou and obtained high-quality sci-ATAC-seq profiles for 1,295 visceral muscle cells. Highlighting these cells on the UMAP shows that they cluster together with the cells assigned as visceral muscle (Figures 1F, orange and 1D, purple). Second, known marker genes for both lineage specification and differentiation show high accessibility in the expected muscle populations at the correct stages (Figures 1G and S2G), including *tinman* (*tin*), *pannier* (*pnr*), and *Doc3* (*Doc3*) in the cardiac muscle;

CellPress
OPEN ACCESS

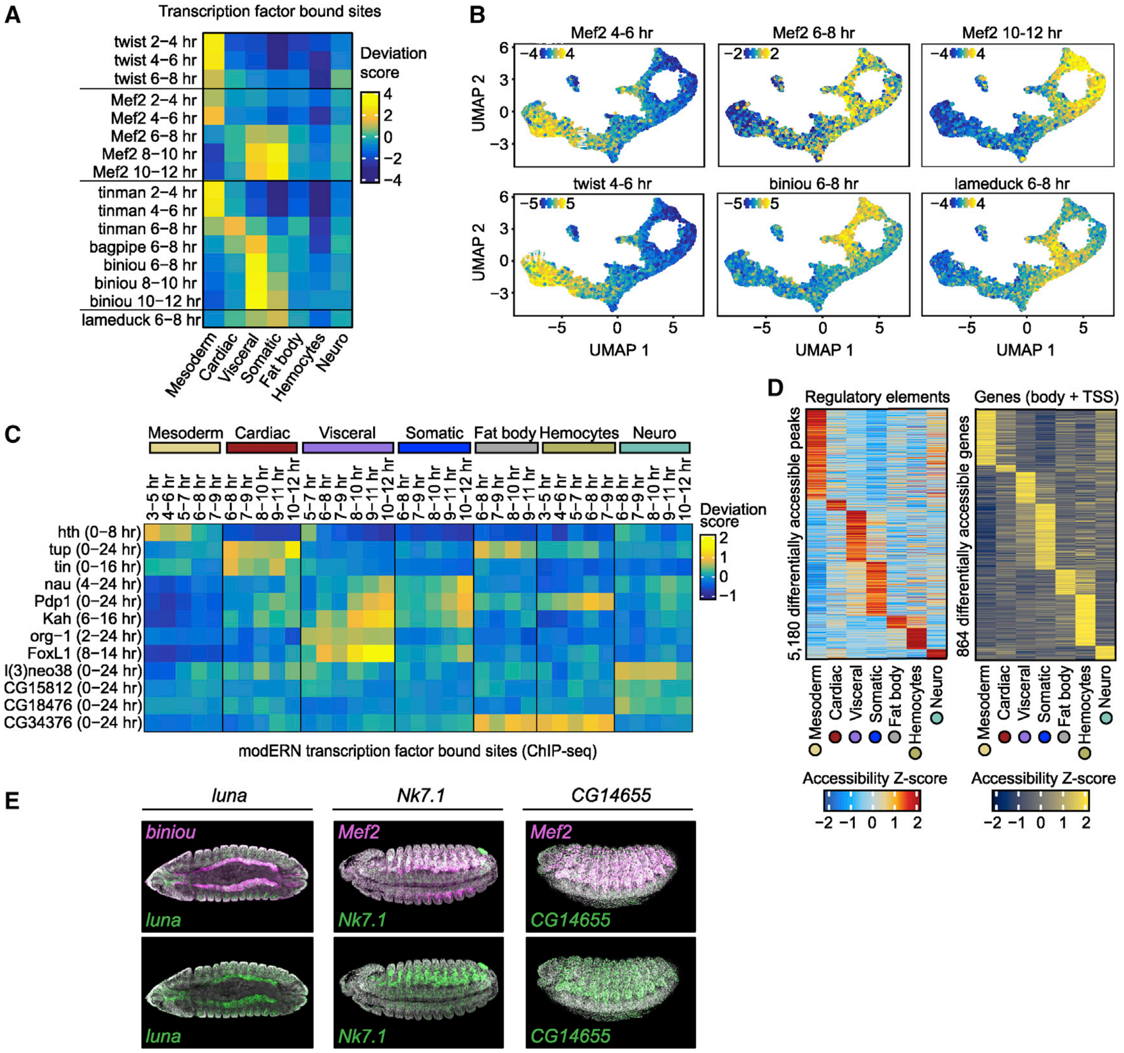

Figure 2. Dynamic changes in the sci-ATAC atlas reflect dynamic transcription factor occupancy and identifies both the regulators and the enhancers in each lineage

(A) Heatmap of accessibility deviation scores for TF-bound regions (from bulk ChIP data) per cell population.

(B) UMAPs with cells colored by accessibility deviation score for the indicated TF using bulk ChIP data from specific time points.

(C) Heatmap of accessibility deviation scores by cell population and developmental time (hours) for sites occupied by selected TFs (ChIP data from modERN). Time points with <5% of cells of a given population were excluded.

(D) Heatmaps of average accessibility (Z score) at differentially accessible ATAC-seq peaks at regulatory elements (left) and genes (right) in each cell population.

(E) Embryos showing the expression of *luna* in the visceral muscle, *Nk7.1* and *CG14655* in the somatic muscle; *in situ* hybridization of the gene (green), and a tissue marker (magenta, *biniou* for visceral muscle, *Mef2* for somatic muscle); DAPI-stained nuclei (gray).

bagpipe (*bap*), *biniou* (*bin*), and *Shaker cognate 1* (*Sha1*) in the visceral muscle, and the *muscle-specific protein 300 kDa* (*Msp300*) and the contractile proteins *Tropomyosin 1* (*Tm1*) and *Myosin heavy chain* (*Mhc*) in differentiated somatic muscle.

Dynamic changes in single-cell chromatin accessibility reflects dynamic transcription-actor binding and identifies new regulators and enhancers in each lineage

To resolve TFs likely responsible for these regulatory changes, we first examined dynamic changes in accessibility overlapping regions bound by muscle-specific factors by calculating TF deviation scores (Schep et al., 2017). These inferred TF activities match the expected patterns for each TF (Figures 2A and 2B). For example, *tinman* is broadly expressed throughout the trunk mesoderm at early stages before being restricted to the dorsal mesoderm and cardiac muscle at later stages (Azpiazu and Frasch, 1993; Yin and Frasch, 1998). Concordantly, Tinman bound sites from bulk ChIP data (Liu et al., 2009; Zinzen et al., 2009) at 2–4 and 4–6 h show high accessibility in cells in the early mesoderm, while Tinman 6–8 h ChIP peaks are restricted to the cardiac cells (Figure 2A). Similarly, Biniou and Bagpipe, the two factors required for visceral mesoderm specification (Azpiazu and Frasch, 1993; Zaffran et al., 2001), show specific activity in cells in the visceral-muscle lineage (Figure 2A). Cells with open

chromatin sites bound by the panmuscle factor Mef2 at either 2–4, 6–8, or 10–12 h of development (Sandmann et al., 2006) display concordant dynamic changes in accessibility over time, being more accessible at early, mid, and late points in our single-cell time course, respectively (Figure 2B, upper). Similar temporal specific activity is seen for cells with regions overlapping twist binding at 4–6 h and Biniou and Lame-duck binding at 6–8 h (Figure 2B, lower) (ChIP data from Cunha et al., 2010; Jakobsen et al., 2007; Sandmann et al., 2007a; Zinzen et al., 2009). Taken together, this indicates that our single-cell atlas accurately recapitulates the underlying temporal and spatial patterns of TF binding during mesoderm development.

To explore this further and identify new potential regulators, we integrated TF occupancy data for 280 factors (Kudron et al., 2018). These bulk ChIP-seq data were generated from whole embryos collected over large time windows spanning either half or all of embryogenesis, and thereby averages signal over different cell types and time points. Nevertheless, the high resolution of our single-cell data could resolve both the cell type and rough time window of occupancy of several factors (Figure 2C). For example, resolving whole-embryo ChIP data for Tinman and Tailup to the cardiac muscle (Azpiazu and Frasch, 1993; Tao et al., 2007; Zmojdzian and Jagla, 2013), Nautilus and Pdp1 to the somatic and visceral muscle (Abmayr and Keller, 1998; Lin et al., 1997), and Org-1 and FoxL1 to visceral muscle (Hanlon and Andrew, 2016; Schaub and Frasch, 2013), consistent with the role of these TFs in the corresponding cell types. Nau is a good example of refining the temporal-window—the ChIP was performed on whole embryos spanning almost all of embryogenesis (4–24 h)—however, integration with our single-cell time course resolved the time window to 10–12 h and the tissue to the somatic and visceral muscle (Figure 2C).

We next used this high-resolution atlas to identify new genes and enhancers that are differentially accessible in a specific muscle subpopulation (Figure 2D; Table S2). Of the 5,180 differential peaks (representing 36% of all tested peaks), 78% (4,027/5,180) are distal from an annotated promoter and likely enhancers. In agreement with this, 20% (790/4,027) overlap previously characterized embryonic enhancers *in vivo*, suggesting that many of the other 80% are also likely enhancers. Remarkably, 19% (752/4,027) of distal differential elements were not discovered in our previous whole-embryo shotgun sci-ATAC-seq (Cusanovich et al., 2018a) or bulk tissue-specific DNase-seq (Reddington et al., 2020) studies (Figure S2H), thereby increasing the discovery of mesoderm/muscle-specific regulatory elements.

In addition, 864 genes are differentially accessible across their gene body between cell types (Figure 2D, right; Table S2), which can serve as a proxy for changes in gene expression. Many of these are components of signaling pathways or TFs, including known regulators of mesoderm/muscle development. For example, the transcription factors *pnr, Doc3*, *tup*, and *apt* in the cardiac; *bap*, *bin*, *H2.0*, and *hand* in the visceral; and *Pdp1* and *cf2* in the somatic muscle (Table S2). In addition, we uncovered many new potential regulators in specific tissues, including *luna*, *Nk7.1*, and *CG14655*. Our sci-ATAC data predict that *luna* is expressed during visceral-muscle development and *Nk7.1* and *CG14655* during somatic muscle development. *In situ* hybridization of each gene with a mesoderm/muscle marker confirms the expression of all three TFs in the tissues predicted by their differential accessibility (Figure 2E). Concordantly, a function for *CG14655* during flight-muscle development was recently reported (Meiler et al., 2021).

Dynamic changes in regulatory elements are sufficient to reconstruct diverse lineage trajectories

We next exploited the continuous temporal resolution of our time course to reconstruct regulatory trajectories for three muscle lineages, starting from unspecified mesodermal cells (Figure 3A, yellow dot). Ordering cells along pseudotime revealed extensive and dynamic temporal changes in accessibility for both regulatory elements and genes along each lineage's trajectory (Figure 3B; Table S3). The loci of many upstream identity genes change in accessibility (in both directions) as the development of each lineage progresses. This includes *lmd*, *kah*, *NK7.1*, *Pdp1*, *tx*, and *nau* (*dMyoD*) in the somatic trajectory, in addition to more downstream effector genes required for differentiated muscle function (*Mhc*, *Mlc*, and *Tropomyosin*). Cardiomyocytes are specified by a highly conserved set of TFs from flies to humans (Davidson and Douglas, 2006), including members of the NKx2.5 (*tinman* [*tin*] in *Drosophila*), GATA (*pannier* [*pnr*]), T-box (*Dorsal corss-3* [*Doc3*]), and islet 1 (*tailup* [*tup*]) TFs. The dynamic usage of all factors and many more are observed along the cardiac lineage (Figures 3B and 3D). Both the somatic and visceral muscles are formed from two populations of cells—founder cells (FCs), which give the muscle its identity, and fusion-competent myoblasts (FCMs), which fuse to FCs during differentiation to form a multinucleated syncytium (Deng et al., 2017; Lee and Chen, 2019). We observed two trajectories for the visceral-muscle lineage (Figure 1D, purple cells). Starting from common precursors, one lineage branches toward the somatic body wall muscle (Figure 1D (ii)), while the other has a distinctive lineage that remains separate from the rest of the muscle populations (Figure 1D (i)).

To explore the visceral-muscle lineages further, we combined all annotated visceral-muscle cells from the Mef2+ time course with the Bin+ FAC sorted cells and reclustered these 4,187 visceral-muscle cells separately, revealing a more complex multi-branched structure (Figure 3C). The visceral muscle represents a collection of muscles with different developmental origins (Figure 3C, embryo scheme). The FCs and FCMs of the circular trunk visceral muscle (CVM) are specified at stages 10 and 11 (6–8 h), after which they migrate laterally and undergo myoblast fusion (stage 12) to form a continuous muscle that encloses the gut (Lee et al., 2006). The longitudinal VM (LVM) is formed from FCs in the caudal mesoderm toward the end of the embryo, which migrate on top of the circular VM, using it as a scaffold (Zaffran et al., 2001). Our single-cell trajectory captures these diverse origins and the temporal delay of the LVM development. The main branch (Figure 3C, cluster 0) consists of both FCs and FCMs, as seen by their enrichment in markers for visceral-muscle FC specification (including *Alk*, *numb*, *bap*, and *bin*) and FCM specific genes (*lmd* and *sns*) (Figure 3D). A subset of cells progress to a more differentiated state (Figure 3C, moving from cluster 0 → 1 → 2), expressing specific myoblast-fusion genes in the intermediate state before differentiating to contractile muscle, indicated by the expression of sarcomere protein genes such as *MHC*, *Tmod*, *Tm1*, and *Tm2* (Figure 3D).

CellPress
OPEN ACCESS

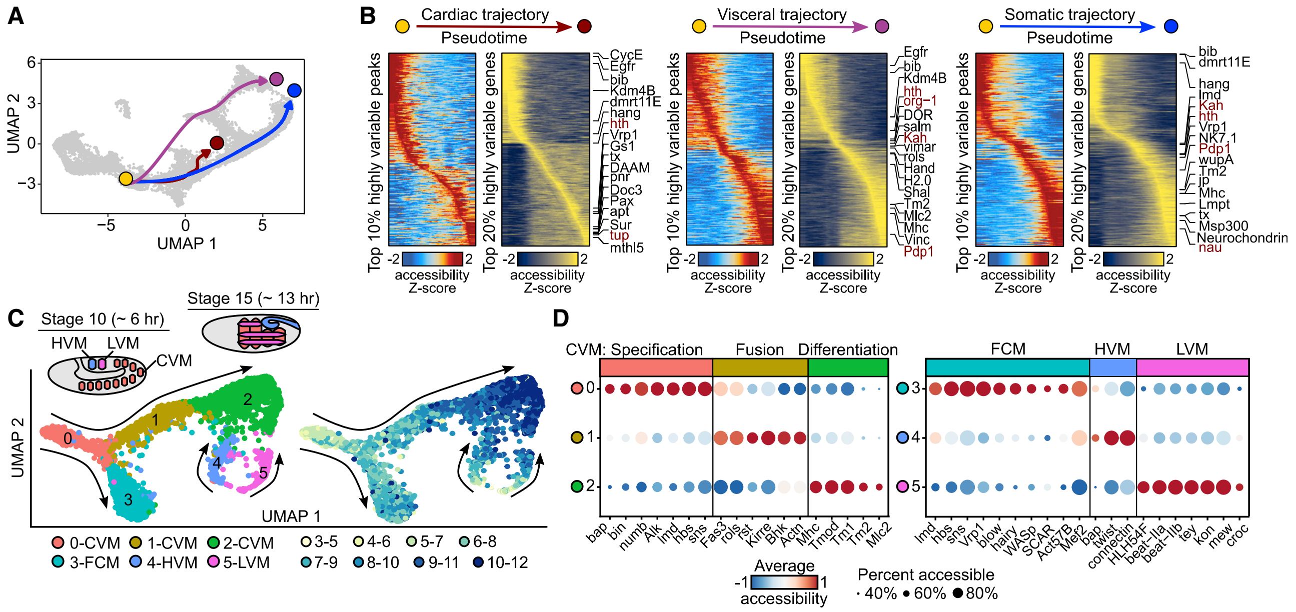

Figure 3. Single-cell regulatory changes can reconstruct the developmental trajectories and identify the TFs and enhancers involved

(A) Inferred cardiac (red), visceral (pink), and somatic (blue) developmental trajectories on UMAP starting from a common point in the unspecified mesoderm (yellow dot).

(B) Heatmaps of ATAC-seq peaks and genes accessibility through pseudotime for each trajectory. Top 10% and 20% most highly variable peaks and genes are shown. TFs identified in Figure 2C, indicated in red.

(C) Top: schematic representing the development of the major visceral-muscle (VM) subtypes. CVM, circular VM; FCM, fusion-competent myoblasts; LVM, longitudinal VM; HVM, hindgut VM. Bottom: UMAP of the reclustered VM population (purple cells from Figure 1D), colored by Seurat cluster (left) and developmental time point (hours) (right).

(D) Marker-gene accessibility for CVM (left) and other VM subtypes (right); color scale indicates gene average accessibility (Z score); dot size the percentage of cells in which the gene is accessible.

Given the timing of these transitions and the presence of *bap* (a TF not expressed in LVM), this likely reflects circular VM development. The LVM and hindgut VM (HVM) branches have different developmental origins, as expected, and are enriched in longitudinal (cluster 5) and hindgut (cluster 4) visceral-muscle markers (Bae et al., 2017; San Martin and Bate, 2001) (Figure 3D). The precursor population has a second branch (Figure 3C, cluster 0 → 3), expressing a broader set of myoblast-fusion genes (Figure 3D), perhaps representing a more mature or more diverse FCM population. Some FCMs were proposed to remain unfused and wait for the LVM founder cells to migrate over the circular VM and then fuse during late stage 12 and stage 13 (~10–12 h) (Klapper et al., 2002). Our results support this and suggest that a proportion of these cells may also fuse to the overlying somatic-muscle FCs, given their expression of *Vrp1/wip* and *wasp*, two genes specific to myoblast fusion of the somatic muscle (Rudolf et al., 2014), and their progressive acquisition of a chromatin state similar to the somatic muscle (cluster 3 overlaps branch (ii) in Figure 1D).

Using single-nucleus genotyping to systematically profile homozygous mutants from mixed embryo populations

To determine the functional impact of TF mutants on both the developmental trajectories of cells and their regulatory programs, we applied sci-ATAC-seq to loss-of-function mutant embryos of four mesodermal TFs and assessed the cells' behavior by integrating them with the wild-type developmental trajectory (Figure 1D). Myocyte enhancer factor 2 (*Mef2*) is essential for myoblast fusion and terminal differentiation of all muscle types (Figure 4A). *Tinman* (Nkx2-5) is essential for the subdivision of the dorsal mesoderm into cardiac and visceral muscle, through the activation of *bagpipe* (NKx3-2), which initiates *biniou* (FoxF2) expression and the visceral-muscle lineage. Although the occupancy of each TF has been examined in bulk (Jakobsen et al., 2007; Junion et al., 2012; Liu et al., 2009; Sandmann et al., 2006, 2007a; Zinzen et al., 2009), their contribution to enhancer accessibility and to an individual cell's state, remains unknown.

Recessive lethal mutants in animal models must be maintained as heterozygotes; therefore, only 25% of offspring embryos are homozygous for the mutation of interest. Here, we first developed an easy and generalizable approach to profile single-cell genomic measurements from embryos of mixed genotypes (Figure 4B). Rather than genotyping and hand sorting homozygous mutant embryos, we retained all embryos representing a pool of three genotypes (homozygous loss-of-function mutant, heterozygous, and homozygous nonmutant) (Figure 4B) and performed sci-ATAC-seq on their dissociated pooled nuclei. In *Drosophila*, such heterozygous mutants are maintained over balancer chromosomes (Miller et al., 2016, 2018). We sequenced the genetic background of the loss-of-function mutants (this study, STAR Methods) and the balancer chromosome (Ghavi-Helm

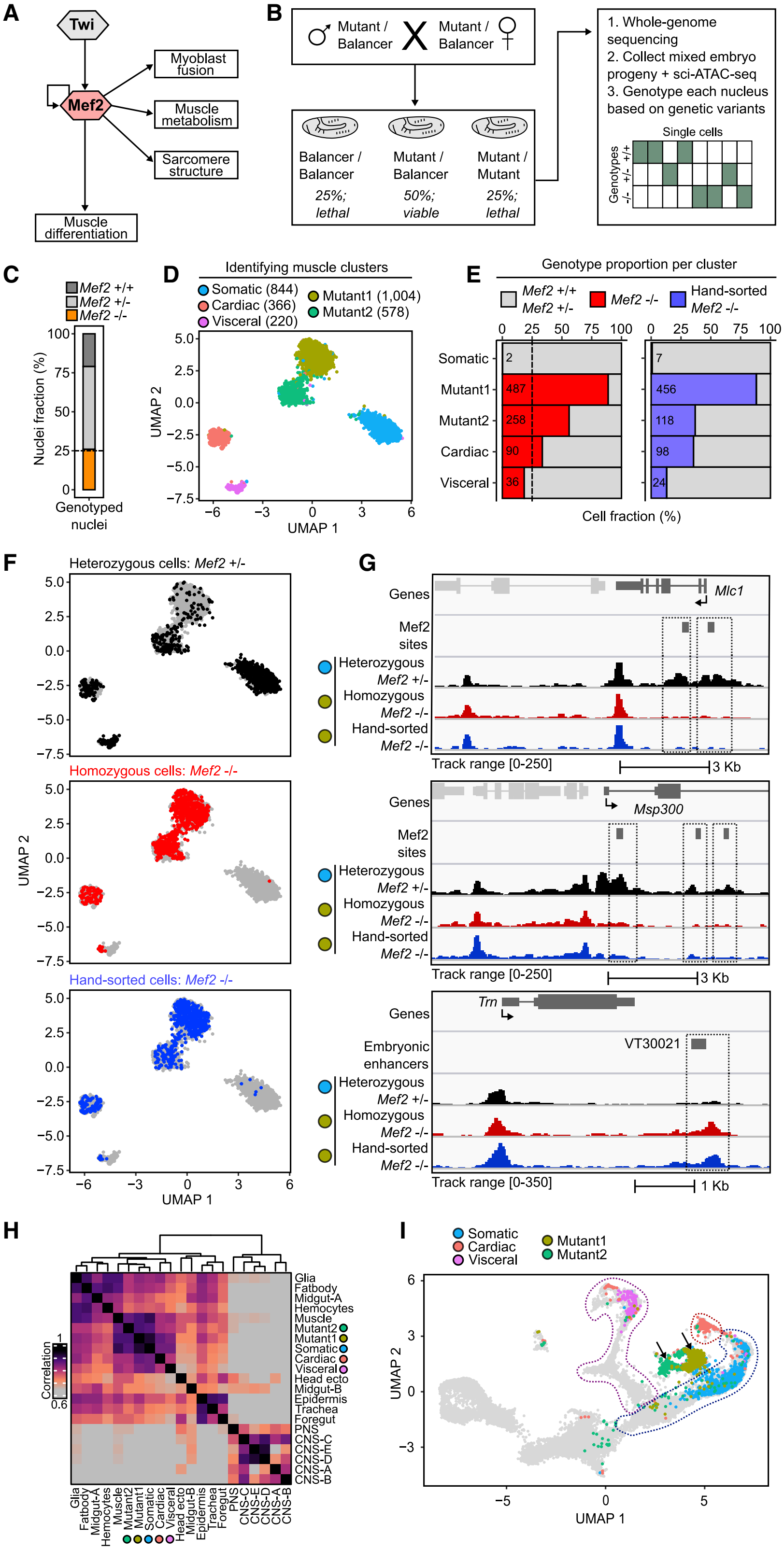

Figure 4. Loss of the transcription factor *Mef2* leads to a new cell state

(A) Simplified schematic of Mef2 functions.

(B) Overview of the nuclear *de novo* genotyping strategy. Pooled embryos of mixed genotypes are dissociated and used for sci-ATAC-seq. Each nucleus is digitally genotyped based on informative genetic variants in scATAC-seq reads.

(C) Proportion of nuclei assigned to each genotype. The expected proportion of *Mef2* homozygous (−/−) nuclei is 25%, indicated by the dashed line.

(D) Muscle clusters in the *Mef2* dataset visualized by UMAP. Cells colored by their inferred cell types.

(E) Left: proportion of digitally genotyped *Mef2* −/− mutant cells (red). Right: proportion of *Mef2* −/− mutant cells from hand-sorted embryos (blue). The heterozygous (*Mef2*/balancer (+/−) and homozygous balancer cells indicated in gray.

(F) Same as (D), *Mef2* +/− cells highlighted in black, digitally genotyped *Mef2* −/− cells in red, and hand-sorted *Mef2* −/− cells in blue.

(G) Pseudobulk accessibility tracks for *Mef2* heterozygous (+/−) cells from the somatic cluster (black) and *Mef2* homozygous (−/−) cells digitally genotyped (red) or hand sorted (blue) from the Mutant1 cluster. Dot color (left) indicates the cluster from (D).

(H) Pearson correlation matrix of clusterwise accessibility for muscle and mutant clusters in (D) with diverse cell clusters from whole embryos (Cusanovich et al., 2018a).

(I) UMAP visualization of the muscle populations identified in (D), including Mef2 +/− and −/− cells, co-clustered with the wild-type muscle time course from Figure 1D. Cell clusters in (D) (*Mef2* embryos) are plotted on the wild-type time course (gray). The somatic, cardiac, and visceral populations from the wild-type time course are enclosed by blue, red, and purple dashed lines, respectively. Note, *Mef2* −/− clusters 1 and 2 (black arrows) are located close to the somatic muscle (blue) but off the wild-type trajectory.

et al., 2019), allowing each nucleus to be genotyped based on informative SNPs in the sci-ATAC reads. In contrast to standard allelic imbalance studies, this requires relatively few informative reads per nucleus, as described below.

The characterized mutations of these TFs were generated over 20 years ago and will have accumulated additional mutations that could impact chromatin accessibility independently of the TFs' function. To circumvent this, we first used CRISPR-Cas9 editing with single-stranded oligo donors (ssODNs) (Gratz et al., 2015) to recreate the characterized loss-of-function mutations for each factor in a common isogenic genetic background (Figure S3A; Table S4; STAR Methods). These new alleles were sequence verified and recapitulate the expected characterized muscle phenotypes (Figure S3B). As essential factors, they are homozygous lethal, and, importantly, our new alleles do not complement the characterized loss-of-function allele when placed in *trans*, confirming that the lethality is due to the mutation of the TF and not a CRISPR off-target effect.

To phenotype mutants (Figure 4B), we collected staged embryos from the heterozygous adults (mutant^{CRISPR}/balancer chromosome), which were formaldehyde fixed and processed for sci-ATAC-seq. The dissociated nuclei thereby come from a pool of F1 embryos which represent 25% homozygous loss-of-function mutant/mutant, 50% heterozygous mutant/balancer, and 25% homozygous balancer/balancer. After sci-ATAC-seq, each nucleus was genotyped *de novo* based on the fraction of reads mapping to the mutant or balancer chromosomes. The genotype assignment is based on over 450,000 genetic variants (Figure S4A) between the balancer and the mutants genetic background. With a median of roughly 1,000 variants covered per cell, we could genotype 99.9% of all theoretically assignable nuclei (Figure S4B) with high confidence (>0.9 posterior probability) (Figure S4C). The genotype assignments were very robust, being 98% identical when performing the assigments using two sets of variants identified with a stringent or a more lenient filtering threshold (Figure S4D).

Our *de novo* single-nucleus genotyping approach has a number of advantages for single-cell profiling of mutants. It eliminates the need, and associated experimental time, to hand select embryos of the correct genotype and is therefore faster and more reliable. Profiling nuclei from homozygous and heterozygous siblings in the same experiment has an additional advantage to aid in batch correction. As the heterozygous nuclei are essentially wild type, they can be used to align mutant data from the same batch to the wild-type reference trajectory, avoiding "over fitting" by batch aligners of biologically real mutant phenotypes.

Loss of the transcription factor *Mef2* leads to a new cell state

Mef2 regulates differentiation of all major muscle types (Bour et al., 1995) (Figure 4A). To determine the functional impact of Mef2 on chromatin accessibility and mesodermal cell fate, we performed sci-ATAC-seq on *Mef2* mutant embryos (a pool of homozygous and heterozygous) at 10–12 h (mainly stage 13) during the initiation of terminal muscle differentiation in the somatic and visceral muscle. Our *de novo* genotyping strategy assigned the expected proportion of profiled nuclei as homozygous mutant (expected: 25%, observed: 26%) (Figure 4C).

As these experiments were performed on whole embryos, we did a first round of clustering to identify muscle cells. The profiled 12,926 cells cluster by cell type, rather than by genotype, revealing 8 broad cell states, including one large muscle population (Figures S5A and S5B; Table S5). Selecting the muscle cells, we then identified genotype and cluster-specific peaks and re-clustered those 2,567 cells (Figure 4D; STAR Methods). This resulted in five cell clusters with distinct chromatin accessibility, three of which could be identified as somatic, cardiac, and visceral muscle (Figure 4D). Digitally genotyped Mef2−/− mutant nuclei are almost completely absent from the somatic-muscle cluster (Figures 4E and 4F) and instead are highly enriched in two additional "muscle clusters," which appear close to, but distinct from, somatic-muscle cells (Mutant1 and Mutant2 clusters). Mutant1 is composed of 89%, while Mutant2 56%, Mef2−/− cells (Figure 4E). This indicates that in the absence of *Mef2*, mesodermal cells are unable to establish the regulatory landscape to become somatic muscle, and instead form a new altered state. Cells in the Mutant2 cluster have lower (~2.2-fold) coverage than the other clusters (Figure S5C). This may represent cells in a more naive state or undergoing apoptosis, although the clustering of these cells might be driven primarily by their lower coverage. The proportion of homozygous mutant cells is also partially reduced in the visceral muscle, while it is largely unaffected in the heart, at these stages (Figures 4E and 4F). These tissue-specific differences likely reflect differences in the timing of differentiation between muscle lineages. Terminal differentiation of the somatic and visceral muscle begins after myoblast fusion at ~10–12 h (stage 13), while it cannot occur in the heart until later stages, after dorsal closure is complete at ~13 h (stage 15).

The somatic and visceral muscle were also the two tissues that displayed the highest relationship between Mef2 binding at 10–12 h and open chromatin signatures (chromVAR scores) in wild-type embryos (Figure 2A), suggesting that such scores are a good indicator of the tissues and time points that will most likely be affected by the removal of a TF. For example, while *Mef2* is also expressed in the cardiac muscle, Mef2-bound regions are generally less accessible compared with the somatic and visceral muscle (Figure 2A) and, concordantly, the proportion of mutant cells remains close to normal in the cardiac muscle (Figure 4E). However, such computational inference from wild-type embryos cannot predict all mutant phenotypes. While the visceral and somatic muscle display similar accessibility (ChromVar scores) at Mef2-bound regions in wild-type embryos (Figure 2A), the mutant analyses revealed clear differences in the response of both tissues to loss of *Mef2*; mutant cells are almost completely absent in the somatic muscle but only partially reduced in visceral (Figures 4E and 4F), indicating a difference in Mef2 dependency for accessibility between these two tissues, which was not evident in wild-type conditions.

To experimentally test the accuracy of our *de novo* genotyping strategy, we hand-sorted homozygous mutant embryos from the *Mef2* mutant based on a GFP-marked balancer chromosome and performed sci-ATAC-seq on these 100% *Mef2*−/− nuclei. The hand-sorted mutant nuclei show the same properties as the genotyped mutant nuclei (Figures 4E and 4F); they are absent from the somatic muscle and accumulate in two mutant clusters—mainly in Mutant1, where 88% of the homozygous mutant cells reside,

similar to the digital genotyping above (Figure 4E). Both the genotyped and hand-sorted homozygous mutant nuclei display the same alterations in chromatin accessibility at individual loci, as shown for two muscle contractile proteins, *Mlc1* and *Msp300* (Figure 4G). Both genes have multiple Mef2-bound regions overlapping open chromatin in cells from the somatic cluster, which are almost completely closed in both the digitally genotyped and hand-sorted Mutant1 cells (Figure 4G). We also observe concordant gains in accessibility at regulatory regions in Mef2−/− cells, including the enhancer VT30021 (Figure 4G), which is embryonically active, but normally not in muscle tissues. This proof of principle indicates that our *de novo* nuclear genotyping strategy correctly assigns homozygous mutant nuclei.

The whole-embryo single-cell data allowed us to explore if *Mef2* mutant cells adopt another cell state, either from within the mesoderm or another germ layer. To assess this, we computed clusterwise accessibility correlations of the mutant clusters against all cell types (both mesodermal and nonmesodermal cell clusters) in embryos at 10–12 h (Cusanovich et al., 2018a) (Figure 4H). Both Mutant1 and Mutant2 are most highly correlated to clusters within the myogenic mesoderm, in particular the somatic muscle, and are clearly separated from the nonmyogenic mesoderm, ectoderm, and endoderm lineages (Figure 4H), indicating that these cells are specified to become muscle, but appear blocked in their development. To determine if they are stuck in an earlier myogenic state, we combined the mutant cell data (combining the digitally genotyped and hand-sorted mutant cells, given that they appear identical) and the heterozygous cells with all cells in our wild-type reference trajectory (Figure 1D) and reclustered the data (Figure 4I). The heterozygous cells (the wild-type clusters in Figure 4D) behave indistinguishably from the reference cells, falling within the expected wild-type populations on the trajectory. In contrast, Mef2−/− Mutant1 and Mutant2 cells cluster separately, off the wild-type muscle trajectory, but roughly at the appropriate “temporal” time point (Figure 4I). If these cells were blocked in their developmental progression, we would expect them to cluster on the trajectory at some earlier time point in muscle development, which is not what we observe. To explore this further, we computed clusterwise accessibility correlations of the mutant and muscle clusters for each time point, which confirmed that both Mutant1 and Mutant2 are progressing to the appropriate developmental stage (Figure S5D). This indicates that *Mef2* mutant cells are not simply immature muscle cells but rather have developed a new abnormal “muscle-like” state, which is likely defined by the inactivation or decreased expression of late muscle function genes, combined with the inappropriate activation of nonmuscle enhancers and genes (Figure 4G; Table S6). Taken together, this suggests that Mef2 is not only required as a differentiation factor to regulate the expression of muscle contractile genes, but also to prevent muscle cells from undergoing other cell-state changes.

Loss of *tinman*, *bagpipe*, and *biniou* differentially alters cellular composition

We applied the same approach to three other loss-of-function mutants for TFs involved in the specification of the dorsal mesoderm (*tinman*) and its derived visceral muscle (*bagpipe* and *biniou*) that forms the gut musculature (Azpiazu and Frasch, 1993; Zaffran et al., 2001). These TFs have a hierarchical relationship between them, where Tinman regulates Bagpipe expression at stage 10 (6–8 h), which in turn regulates Biniou expression (Figure 5A). To examine the function of these TFs, we assessed *bagpipe* and *biniou* mutants at 6–8 h of development, which coincides with the initiation of their expression and with the specification of the visceral muscle (stages 10 and 11). As Tinman acts upstream of both *bap* and *bin*, we shifted the time window 1 h earlier (5–7 h; stage 9, 10) to capture these events. For all three mutants, sci-ATAC-seq was performed on a pool of homozygous and heterozygous embryos, as above. Staged *bagpipe* and *biniou* mutant embryos were collected at 6–8 h (late stage 10, mainly stage 11) and the mesodermal population isolated by Mef2 FAC sorting, as in the wild-type trajectory, obtaining high-quality profiles for 6,306 and 5,833 mesodermal cells, respectively. Presorting for the mesodermal population was not possible for *tinman*, as it regulates Mef2 expression. We therefore performed sci-ATAC-seq on whole embryos of *tinman* mutants and performed a first round of clustering to identify 6,786 high-quality mesodermal cells.

To assess the fate of the mutant cells, we directly compared their development with the wild-type trajectory by co-clustering the combined mutant data, representing 18,925 homozygous and heterozygous cells, together with our wild-type mesoderm time course, correcting for batch-level effects (Korsunsky et al., 2019) (STAR Methods; Figures S5E–S5H). Reclustering and reannotation of this joint dataset (Table S5), representing 40,232 cells, revealed a structure that is generally consistent with the wild-type trajectory (Figure 5B). Nuclei from heterozygous mutant cells (+/−) for *tinman* are present in the cardiac (cluster C1) and early visceral-muscle clusters (clusters V1–3, plus V5 and V6) (Figure 5C, middle, black). Similarly, *bagpipe* and *biniou* heterozygous mutant cells are present in the visceral mesoderm, spanning both branches, and extending to later stages of embryogenesis (clusters V1–4, plus V5 and V6) (Figures 5D and 5E, middle, black).

Examining the homozygous mutant nuclei (−/−) revealed that, in contrast to *Mef2*, the proportion was significantly lower than the expected 25%, representing 15%, 19%, and 17% for *tinman*, *bagpipe*, and *biniou*, respectively (Figures 5C–5E, left). This indicates that a proportion of homozygous mutant cells is not maintained and likely undergos apoptosis as they cannot progress in their development. The trajectories of the remaining mutant cells (−/−) are very different from their heterozygous siblings; *tinman* −/− cells are completely absent from the cardiac lineage and late-stage visceral muscle (V3–V6), with few remaining cells in the early visceral-muscle clusters (Figure 5C, middle, red; V1 and V2). Moreover, there is a significant reduction of homozygous mutant cells in late mesoderm stages (clusters M4 and M5), which likely represents the dorsal mesoderm. Similarly, the *bagpipe* and *biniou* homozygous mutant nuclei are absent from visceral-muscle clusters at later stages of development (clusters V2 and V3, Figures 5D and 5E, middle, red). The early visceral cells (cluster V1) are more prominently affected in *tinman* mutants and to a lesser extent in *bagpipe* and *biniou* mutants, reflecting the hierarchical position of these TFs with Tinman acting upstream of both factors. These findings indicate that the circular VM cells are initially specified in *bagpipe* and *biniou* mutant embryos but are blocked from further expansion and differentiation, resulting in a loss of the VM at later stages (clusters V2 and V3). Interestingly, the hindgut and longitudinal visceral muscles

CellPress
OPEN ACCESS

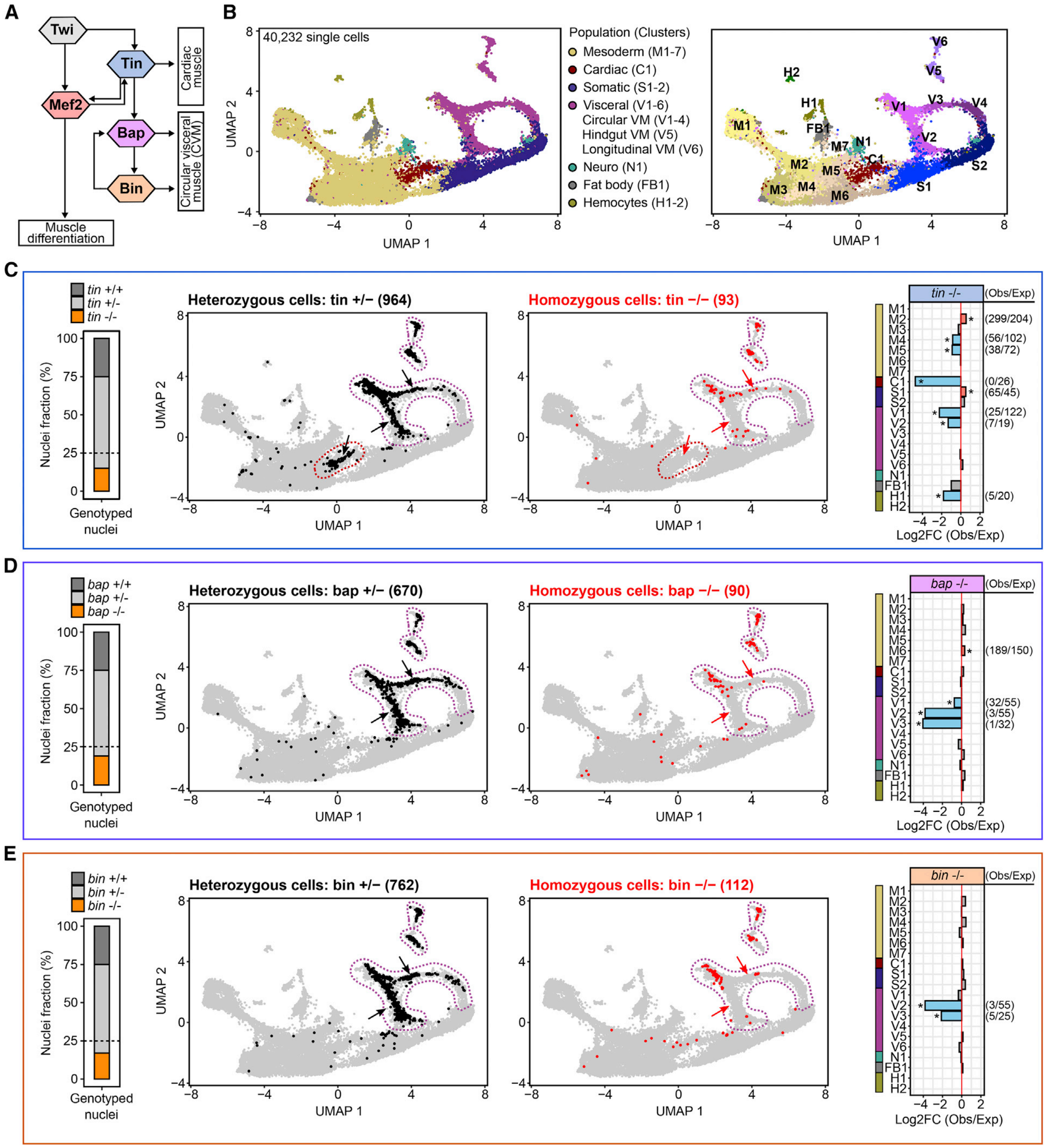

Figure 5. Loss of *tinman*, *bagpipe*, and *biniou* differentially alters cellular composition
(A) Simplified schematic of the mesoderm regulatory network dissected from genetic studies.
(B) UMAP visualization of the wild-type sci-ATAC time course reclustered with single-cell data from *tinman (tin)*, *bagpipe (bap)*, and *biniou (bin)* heterozygous (+/−) and homozygous (−/−) embryos. Each dot represents a single cell, colored by cell-type annotation (left) and by cell cluster (right).
(C) Left: proportion of nuclei assigned to each genotype. The expected proportion of homozygous −/− mutant nuclei is 25%, indicated by the dashed line. Middle: same as (B), where cells from *tin* embryos (5–7 h) are colored by genotype (heterozygous tin +/− cells in black; homozygous *tin* −/− cells in red) and the cardiac (dark red in B) and visceral (purple in B) muscle populations highlighted with dashed lines. The cell numbers highlighted in the UMAPs are indicated in parenthesis. Only cells belonging to the cardiac and visceral populations are plotted. Right: Log_2 fold-change of observed versus expected *tin* −/− cells in each cell cluster (shown for clusters with >100 cells). Asterisks indicate clusters with a significant (Fisher's exact test; p value < 0.05) over- (red bars) or underrepresented (light blue bars) of *tin* −/− cells, numbers of observed/expected cells are indicated (right).
(D) Same as (C), highlighting cells from *bagpipe* heterozygous and homozygous embryos (6–8 h): *bap* +/− (black) and *bap* −/− (red).
(E) Same as (C), highlighting cells from *biniou* heterozygous and homozygous embryos (6–8 h): *bin* +/− (black) and *bin* −/− (red). Arrows in UMAPs (C and D) point to heart (C) and visceral muscle (C and D) (outlined by dased lines), highlighting differences between heterozygous (black) and homozygous (red) populations.

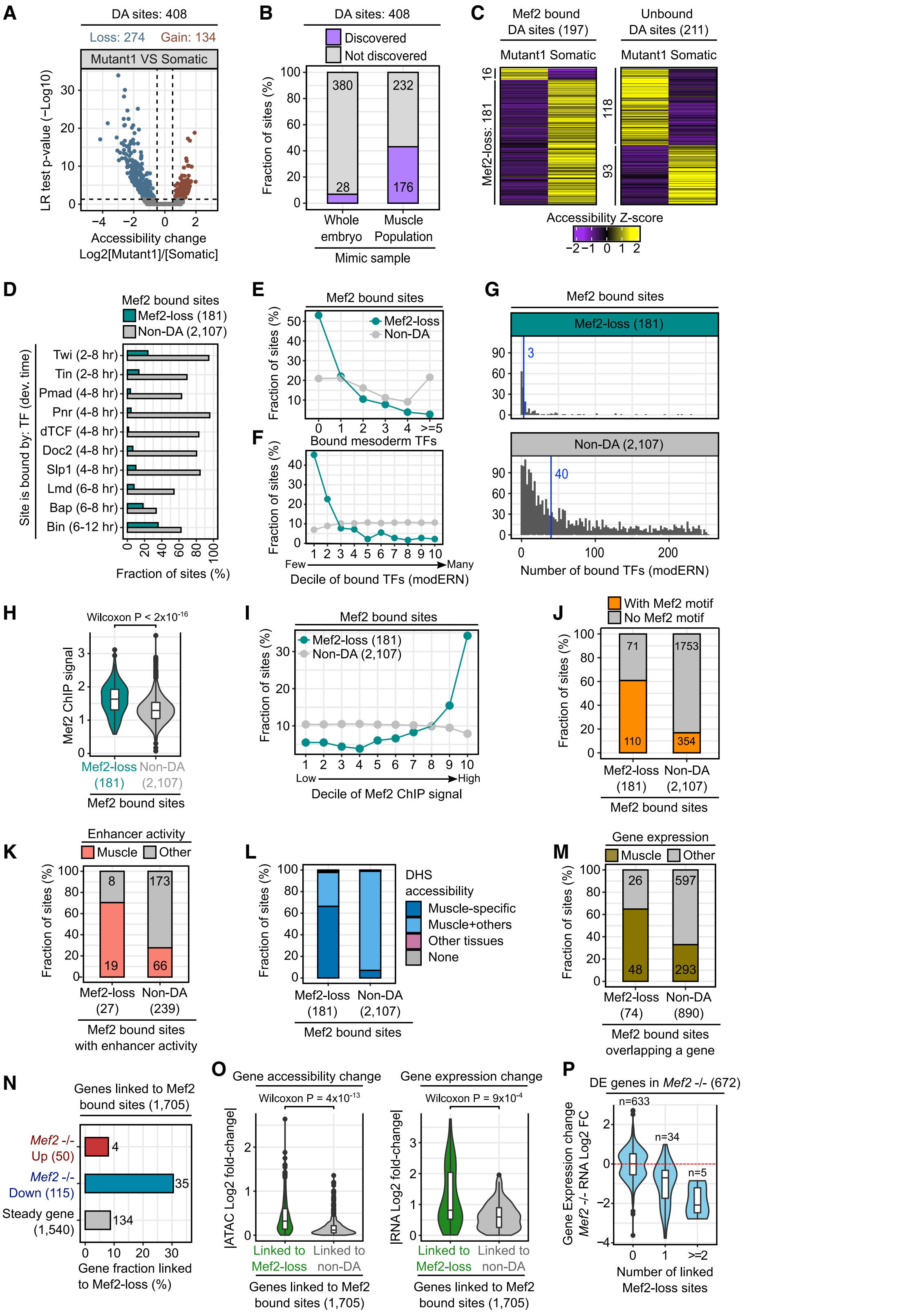

Figure 6. Mef2 functions as an activator, regulating chromatin accessibility at its high-affinity sites

(A) Volcano plot of 408 differentially accessible (DA) sites. Log_2 fold-change in chromatin accessibility (x axis), showing an increase (positive values) or decrease (negative values) in Mutant1 compared with somatic-muscle cluster; y axis indicates Bonferroni corrected p value ($-Log_{10}$ scale).

(legend continued on next page)

appear largely unaffected in all three mutants (clusters V5 and V6, Figures 5C–5E), reflecting their different developmental origin.

This molecular data can therefore phenotype all three mutants *de novo*, identifying the gross phenotypes described by immunostaining of mutant embryos (Figure S3B) (Azpiazu and Frasch, 1993; Zaffran et al., 2001). In addition, our single-cell approach provides more fine-grained quantitative information on the proportion of missing cells at a given stage (Figures 5C–5E, right), in addition to revealing more subtle phenotypes not previously observed, including a gain of mutant cells in other muscle lineages. For example, there is a significant overrepresentation of *tinman* mutant cells in the early mesoderm (cluster M2) and in the somatic lineage (cluster S1) (Figure 5C, right). The removal of these TFs thereby not only results in a loss of tissue (one cell fate) but also a more subtle gain of cells dispersed in other tissues from different mesodermal trajectories, highlighting the plasticity of cell fates within the myogenic mesoderm.

Mef2 is required for chromatin accessibility at its high-affinity sites and for gene expression

Applying single-cell ATAC-seq to TF mutants in the context of a developing embryo allowed us to explore the extent to which such single-cell data can discern regulatory properties of the TF or its enhancers. As a proof of principle, we focus on the Mef2−/− mutant cell cluster (Mutant1), as it contains the highest number of mutant cells (943 cells). The somatic-muscle cluster is the closet cell type to Mutant 1 (Figure 4H). Of the 8,725 accessible regions in both cell clusters, 408 have significant differential accessibility (DA) in Mef2−/− mutant cell ($\log_2$ fold-change > ±0.5, Bonferroni corrected p value < 0.05) (Figure 6A; Table S6). The majority of DA sites have reduced accessibility (67% [274/408]) and reduced sites often have a larger fold-change (Figure 6A). Mef2-responsive sites are generally more gene distal, compared with unchanged sites (Figure S6A) and are overrepresented in muscle enhancers: *Mef2* −/− DA regions more frequently overlap (1) characterized muscle enhancers (Figure S6B), and (2) two large collections of putative muscle enhancers defined by ChIP (Zinzen et al., 2009) (Figure S6C) and DNase-seq of FACS-sorted muscle cells (Reddington et al., 2020) (Figure S6D), compared with non-DA regions.

Ninety percent of the 8,725 accessible regions in either the somatic and/or Mutant1 cluster were identified in bulk DNase-seq from FACS-sorted muscle cells purified at different stages of embryogenesis (Reddington et al., 2020) (Figure S6D). While this highlights the quality of our sci-ATAC-seq data, having such information at a single-cell resolution goes beyond the detection of regulatory regions, as it reveals enhancer usage along developmental trajectories of specific cell types (as shown above) and enables a detailed analysis of the functional input of TFs to enhancers (discussed below). In addition, single-cell data have enhanced the sensitivity to identify regions that change in mutant embryos and enhanced the precision to uncover the cellular context that is susceptible to that change. To demonstrate this, we repeated the analysis in Figure 6A by testing for differential accessibility between nonmutant and mutant cells across the whole embryo and across the whole muscle population, in order to mimic samples profiled by bulk ATAC-seq in either whole embryos or FACS-purified muscle, respectively. Of the 408 DA sites in *Mef2* mutants, only 7% (28) and 43% (176) are identified as significantly changed (using the same parameters as Figure 6A) compared with whole-embryo and muscle mimic samples, respectively (Figure 6B). Our single-cell approach therefore has enhanced sensitivity to reveal regulatory changes that are not discoverable by traditional approaches, unless complex and extensive FAC sorting is used.

To explore the 408 DA sites further, we first categorized them into Mef2-bound and -unbound sites, using bulk Mef2 ChIP data at multiple time points of embryonic development (Zinzen et al., 2009). Almost half of the DA regions (48%, 197/408) are bound by Mef2 at this stage or earlier in embryogenesis. Mef2-bound DA sites almost exclusively lose accessibility (Figure 6C), consisting of 66% of all DA sites with reduced accessibility (Figure S6E). In contrast, regions that gain accessibility are generally not bound by Mef2 (Figure 6C) and involve regions less related to muscle function (Figures S6F–S6H).

Removal of Mef2 affects the accessibility of only a specific subset (15%) of all Mef2-bound regions at 10–12 h. Mef2-bound sites that are sensitive to, or resistant to, Mef2 removal could depend on either their extent of co-occupancy by other factors or on the affinity of Mef2 binding. To distinguish between these

(B) Proportion of 408 DA sites discovered as DA comparing mutant and nonmutant cells in whole-embryo or muscle-mimic samples, using logistic regression.
(C) Heatmaps of chromatin accessibility at the 408 DA sites, split by those bound by Mef2 during embryogenesis (Mef2-bound, 197 DA sites) or not bound (Unbound; 211 DA sites). Over 90% (181/197) of Mef2-bound regions have reduced accessible in *Mef2* mutant cells (Mutant1).
(D) Fraction of Mef2-bound sites occupied by additional mesodermal TFs for DA sites with reduced Mef2-loss (green) or unchanged (non-DA, (gray) accessibility in *Mef2* −/− cells. TF occupancy was measured by ChIP at the indicated embryonic time.
(E) Fraction of Mef2-bound sites occupied by the indicated number of mesodermal TFs for DA sites with reduced accessibility (Mef2-loss, green) and non-DA sites in *Mef2* mutant cells. X axis value 0 indicates sites bound by Mef2 and no additional mesodermal TF.
(F) Fraction of Mef2-bound sites occupied by increasing numbers of general TFs (from modERN) stratified by decile for DA Mef2 loss and non-DA sites.
(G) Number of TFs (modERN) that occupy DA Mef2-loss and non-DA Mef2-bound sites. Blue line indicates the median number of bound TFs.
(H) Quantification of the Mef2 ChIP signal at DA Mef2-loss and non-DA Mef2-bound sites. Wilcoxon p value indicated.
(I) Fraction of Mef2-bound sites stratified by decile of increasing Mef2 ChIP signal, shown for both DA Mef2-loss (green) and non-DA sites (gray).
(J) Fraction of DA Mef2-loss and non-DA Mef2-bound sites with (orange) or without (gray) a Mef2 motif.
(K–M) Fraction of DA Mef2-loss and non-DA Mef2-bound sites that overlap (K) characterized embryonic enhancers with demonstrated activity in muscle (red) or other tissues (gray), (L) DNase I hypersensitive sites (DHS) accessible in muscle only (muscle-specific), in muscle and other tissues (muscle + others), in other tissues only (other tissues) or that do not overlap a DHS (none) and (M) genes with expression in muscle (brown) or other tissues (gray).
(N) Fraction of genes linked to a DA Mef2-loss site split by gene-expression state (up, down, or unchanged) in *Mef2* −/− embryos.
(O) Absolute ATAC (left) and RNA (right) $\log_2$ fold-change in *Mef2* −/− embryos, split by genes linked to DA Mef2-loss (green) or non-DA (gray) Mef2 sites. Wilcoxon p value indicated.
(P) RNA $\log_2$ fold-change of differentially expressed genes in *Mef2* −/− embryos associated with an increasing number of DA Mef2-loss sites.

two possibilities, we first examined the co-occupancy of ten TFs active in mesoderm. Susceptible sites are generally less frequently occupied by these TFs compared to nonsusceptible sites (Figure 6D). The fraction of DA sites tends to decrease as sites are bound by an increasing number of mesodermal TFs (Figure 6E). Examining the occupancy of a much larger set of TFs, 280 factors from modERN (Kudron et al., 2018) also shows an inverse relationship between differential chromatin accessibility in Mef2−/− cells and the number of bound TFs (Figure 6F): the median number of bound TFs is 3 for the DA class and 40 for non-DA sites (Figure 6G). Therefore, sites that require Mef2 for their accessibility tend to be bound by Mef2 alone or with a small number of other factors, perhaps cooperatively, suggesting that these regions are very Mef2 dependent. To investigate this further, we used the quantitative Mef2 ChIP signal as a proxy for Mef2 affinity. Susceptible (DA) sites have significantly higher Mef2 ChIP signal compared with nonsusceptible (non-DA) sites (Figure 6H). Moreover, the proportion of DA sites steadily increases as the Mef2 ChIP signal increases, going from 5% of DA sites in the lowest to 35% in the highest ChIP quantile (Figure 6I). This indicates that sites bound more strongly by Mef2 are more likely to have reduced chromatin accessibility upon Mef2 removal. Although both classes are occupied by Mef2, susceptible sites have a 2.5-fold enrichment (61% in the DA group versus 17% in the non-DA group, Fisher's exact test p value = 2×10^{-25}) in the presence of a Mef2 motif (Figure 6J). These results indicate that Mef2 is required to establish and/or maintain chromatin accessibility at a large fraction of its high-affinity sites.

Many of these regions overlap characterized (Figure 6K) or putative (Figure 6L) muscle enhancers. The loss of accessibility at these sites may therefore lead to changes in the expression of mesoderm/muscle genes (Figure 6M), which most likely contributes to the mutant phenotype. To examine this, we integrated bulk RNA-expression data from *Mef2* mutant embryos (Sandmann et al., 2006) and looked for genes with a Mef2-bound site in their vicinity (defined as 5 kb upstream and intronic regions). Using this metric, 1,705 differentially expressed genes are associated with at least one Mef2-bound open chromatin region (Table S6). Of these, those with significantly downregulated, but not upregulated, expression ($\log_2$ fold-change > ±0.7, q < 0.05) in *Mef2* −/− mutants are highly overrepresented for a loss or reduction in chromatin accessibility in at least one of their Mef2-bound associated peaks (Figure 6N). Moreover, genes with reduced Mef2-bound sites have significantly stronger changes in both their chromatin accessibility (Wilcoxon p value = 4×10^{-13}) and gene expression (Wilcoxon p value = 9×10^{-4}), compared with genes with unchanged Mef2-bound sites (Figure 6O). Many known Mef2 target genes are among this set, including *Mhc*, *Mlc1/2*, *Tm1*, *Mp20*, *Mlp60A*, and *Msp300*. In addition, their expression changes become more severe with increasing numbers of associated regulatory regions with reduced accessibility (Figure 6P). These findings indicate that Mef2 functions primarily as an activator and as the predominant regulator for the expression of these genes, which in turn likely leads to the muscle defects in *Mef2* mutant embryos. It is also a rare example demonstrating that a single TF can affect the regulation of many genes by having a cumulative effect on their expression through the action of multiple dependent enhancers.

DISCUSSION

Here, we present a general framework to obtain a fine-grained view of TF function at both a cellular and molecular level using a systematic, unbiased approach. Phenotypes of developmental mutants are typically assessed by immunostaining with tissue markers and often described in qualitative and somewhat arbitrary terms. There are many examples where other phenotypes were missed as the tissue was outside the interests or scope of the study, and in some cases, suitable tissue markers were not available because they are downstream of the mutated TF. When they are, translating such coarse-grained tissue defects to the underlying molecular function of the TF remains a challenge, and typically the regulatory input is only assessed by occupancy in wild-type embryos compared with gene-expression changes in the mutant. Here, we show how single-cell regulatory trajectories, obtained by a dense time course of developmental stages, provide a new opportunity to map developmental mutants to much more precise cell states, thereby providing more fine-grained insights into mutant phenotypes. In the four mutants studied here, this approach not only revealed the loss of the expected cell types but could also quantify the proportion of cells lost, pinpoint the development stages, and reveal more subtle phenotypes, such as a gain of some mutant cells in seemingly normal trajectories of other tissues. This highlights the plasticity of mesodermal cell states and also a high degree of canalization to developmental programming, even upon mutation of these essential TFs. This could increase the overall robustness of embryogenesis, for example, by providing an excess of cells that can partly compensate for the loss of others when defects occur. The data provide a rich resource of regulatory changes associated with each step of mesoderm specification and differentiation into different muscle types, which we provide as easy to search, interactive UMAPs for further exploration (http://furlonglab.embl.de/ss/Drosophila-Mesoderm-Chromatin-Accessibility/). Going forward, this approach could be applied to reassess phenotypes and regulatory programs of "classic" developmental mutants and also to uncover phenotypes of completely uncharacterized mutants *de novo*, beckoning a new era for joint cellular and molecular phenotyping.

Limitations of the study

Although the approach presented here can readily identify cellular phenotypes from mutant embryos, it might not be possible to study molecular phenotypes in mutants in cases where specific cell types are not specified or maintained. This limitation could be overcome by using conditional depletion/knockout strategies (Kögler et al., 2021). Dissection of molecular phenotypes using scATAC-seq could also be masked by the binding of other TFs to the same enhancers. Here, using single-cell ChIP against H3K27ac and/or nascent RNA-seq to measure eRNA at single-cell resolution would help, although both approaches are still very challenging to apply to embryos.

STAR★METHODS

Detailed methods are provided in the online version of this paper and include the following:

- KEY RESOURCES TABLE
- RESOURCE AVAILABILITY
  - Lead contact
  - Materials availability
  - Data and code availability
- EXPERIMENTAL MODEL AND SUBJECT DETAILS
  - *D. melanogaster* model
  - Generation of CRISPR strains
- METHOD DETAILS
  - Embryo fixation and nuclear isolation
  - Nuclear staining for FANS of mesoderm / muscle populations
  - Generation of sci-ATAC-seq libraries
  - Immunostaining and *in situ* hybridization
- QUANTIFICATION AND STATISTICAL ANALYSIS
  - Raw data processing and cell assignment
  - Peak calling and generation of bigwig tracks
  - LDA, clustering and UMAP visualization
  - Clustering of the wild-type time course
  - Calculation of gene activity scores
  - Cell type annotation
  - Transcription factor deviation scores
  - Pseudotime analysis
  - Identification of DA peaks and genes for muscle subpopulations
  - Single-nucleus genotyping
  - Clustering of the *Mef2* mutant dataset
  - Clustering of *tinman*, *bagpipe* and *biniou* mutant datasets
  - Differential ATAC peak analysis in *Mef2* mutant embryos

SUPPLEMENTAL INFORMATION

Supplemental information can be found online at https://doi.org/10.1016/j.devcel.2022.01.016.

ACKNOWLEDGMENTS

The authors thank previous and current Furlong lab members for very helpful discussions, in particular James Reddington. This work was technically supported by the EMBL's flow cytometry and Genomics core facilities and fly *Drosophila* injection service and by the public FlyBase and RedFly databases. E.E.M.F. is funded by grants from the Deutsche Forschungsgemeinschaft and European Research Council (ERC advanced grant) agreement 787611 (DeCRyPT).

AUTHOR CONTRIBUTIONS

S.S. and E.E.M.F. conceptualized and planned the study. S.S. performed all experiments and the sci-ATAC-seq computational analysis. M.F. identified genetic variants for the single-nucleus genotyping. T.H. performed the genotyping, with supervision from O.S. S.S. and E.E.M.F. wrote the manuscript with input from all authors. E.E.M.F. supervised the project and provided funding.

DECLARATION OF INTERESTS

Eileen E. Furlong is a member of the *Developmental Cell* editorial advisory board.

Received: June 30, 2021
Revised: November 4, 2021
Accepted: January 26, 2022
Published: February 16, 2022

REFERENCES

Abmayr, S.M., and Keller, C.A. (1998). *Drosophila* myogenesis and insights into the Role of nautilus. Curr. Top. Dev. Biol. *38*, 35–80.

Amini, S., Pushkarev, D., Christiansen, L., Kostem, E., Royce, T., Turk, C., Pignatelli, N., Adey, A., Kitzman, J.O., Vijayan, K., et al. (2014). Haplotype-resolved whole-genome sequencing by contiguity-preserving transposition and combinatorial indexing. Nat. Genet. *46*, 1343–1349.

Argelaguet, R., Clark, S.J., Mohammed, H., Stapel, L.C., Krueger, C., Kapourani, C.A., Imaz-Rosshandler, I., Lohoff, T., Xiang, Y., Hanna, C.W., et al. (2019). Multi-omics profiling of mouse gastrulation at single-cell resolution. Nature *576*, 487–491.

Azpiazu, N., and Frasch, M. (1993). Tinman and bagpipe: two homeo box genes that determine cell fates in the dorsal mesoderm of *Drosophila*. Genes Dev *7*, 1325–1340.

Azpiazu, N., Lawrence, P.A., Vincent, J.P., and Frasch, M. (1996). Segmentation and specification of the *Drosophila* mesoderm. Genes Dev *10*, 3183–3194.

Bae, Y.K., Macabenta, F., Curtis, H.L., and Stathopoulos, A. (2017). Comparative analysis of gene expression profiles for several migrating cell types identifies cell migration regulators. Mech. Dev. *148*, 40–55.

Bodmer, R. (1993). The gene tinman is required for specification of the heart and visceral muscles in *Drosophila*. Development *118*, 719–729.

Bolger, A.M., Lohse, M., and Usadel, B. (2014). Trimmomatic: a flexible trimmer for Illumina sequence data. Bioinformatics *30*, 2114–2120.

Bonn, S., Zinzen, R.P., Girardot, C., Gustafson, E.H., Perez-Gonzalez, A., Delhomme, N., Ghavi-Helm, Y., Wilczyński, B., Riddell, A., and Furlong, E.E.M. (2012a). Tissue-specific analysis of chromatin state identifies temporal signatures of enhancer activity during embryonic development. Nat. Genet. *44*, 148–156.

Bonn, S., Zinzen, R.P., Perez-Gonzalez, A., Riddell, A., Gavin, A.C., and Furlong, E.E.M. (2012b). Cell type-specific chromatin immunoprecipitation from multicellular complex samples using bits-chip. Nat. Protoc. *7*, 978–994.

Bour, B.A., O'Brien, M.A., Lockwood, W.L., Goldstein, E.S., Bodmer, R., Taghert, P.H., Abmayr, S.M., and Nguyen, H.T. (1995). *Drosophila* MEF2, a transcription factor that is essential for myogenesis. Genes Dev *9*, 730–741.

Bravo González-Blas, C., Minnoye, L., Papasokrati, D., Aibar, S., Hulselmans, G., Christiaens, V., Davie, K., Wouters, J., and Aerts, S. (2019). cisTopic: cis-regulatory topic modeling on single-cell ATAC-seq data. Nat. Methods *16*, 397–400.

Briggs, J.A., Weinreb, C., Wagner, D.E., Megason, S., Peshkin, L., Kirschner, M.W., and Klein, A.M. (2018). The dynamics of gene expression in vertebrate embryogenesis at single-cell resolution. Science *360*.

Cannavò, E., Koelling, N., Harnett, D., Garfield, D., Casale, F.P., Ciglar, L., Gustafson, H.E., Viales, R.R., Marco-Ferreres, R., Degner, J.F., et al. (2017). Genetic variants regulating expression levels and isoform diversity during embryogenesis. Nature *541*, 402–406.

Chen, H., Lareau, C., Andreani, T., Vinyard, M.E., Garcia, S.P., Clement, K., Andrade-Navarro, M.A., Buenrostro, J.D., and Pinello, L. (2019). Assessment of computational methods for the analysis of single-cell ATAC-seq data. Genome Biol *20*, 241.

Ciglar, L., Girardot, C., Wilczyński, B., Braun, M., and Furlong, E.E. (2014). Coordinated repression and activation of two transcriptional programs stabilizes cell fate during myogenesis. Development *141*, 2633–2643.

Corces, M.R., Trevino, A.E., Hamilton, E.G., Greenside, P.G., Sinnott-Armstrong, N.A., Vesuna, S., Satpathy, A.T., Rubin, A.J., Montine, K.S., Wu, B., et al. (2017). An improved ATAC-seq protocol reduces background and enables interrogation of frozen tissues. Nat. Methods *14*, 959–962.

Crittenden, J.R., Skoulakis, E.M.C., Goldstein, E.S., and Davis, R.L. (2018). *Drosophila* mef2 is essential for normal mushroom body and wing development. Biol. Open *7*, bio035618.

Cunha, P.M.F., Sandmann, T., Hilary Gustafson, E., Ciglar, L., Eichenlaub, M.P., and Furlong, E.E.M. (2010). Combinatorial binding leads to diverse

regulatory responses: Lmd is a tissue-specific modulator of Mef2 activity. PLoS Genet *6*, e1001014.

Cusanovich, D.A., Hill, A.J., Aghamirzaie, D., Daza, R.M., Pliner, H.A., Berletch, J.B., Filippova, G.N., Huang, X., Christiansen, L., DeWitt, W.S., et al. (2018b). A single-cell atlas of in vivo mammalian chromatin accessibility. Cell *174*, 1309–1324.e18.

Cusanovich, D.A., Reddington, J.P., Garfield, D.A., Daza, R.M., Aghamirzaie, D., Marco-Ferreres, R., Pliner, H.A., Christiansen, L., Qiu, X., Steemers, F.J., et al. (2018a). The cis-regulatory dynamics of embryonic development at single-cell resolution. Nature *555*, 538–542.

Davidson, E.H., and Erwin, D.H. (2006). Gene regulatory networks and the evolution of animal body plans. Science *311*, 796–800.

Davie, K., Janssens, J., Koldere, D., De Waegeneer, M., Pech, U., Kreft, Ł., Aibar, S., Makhzami, S., Christiaens, V., Bravo González-Blas, C., et al. (2018). A single-cell transcriptome atlas of the aging *Drosophila* brain. Cell *174*, 982–998.e20.

Deng, S., Azevedo, M., and Baylies, M. (2017). Acting on identity: myoblast fusion and the formation of the syncytial muscle fiber. Semin. Cell Dev. Biol. *72*, 45–55.

Domcke, S., Hill, A.J., Daza, R.M., Cao, J., O'Day, D.R., Pliner, H.A., Aldinger, K.A., Pokholok, D., Zhang, F., Milbank, J.H., et al. (2020). A human cell atlas of fetal chromatin accessibility. Science *370*, eaba7612.

Farley, E.K., Olson, K.M., and Levine, M.S. (2015). Regulatory principles governing tissue specificity of developmental enhancers. Cold Spring Harb. Symp. Quant. Biol. *80*, 27–32.

Farrell, J.A., Wang, Y., Riesenfeld, S.J., Shekhar, K., Regev, A., and Schier, A.F. (2018). Single-cell reconstruction of developmental trajectories during zebrafish embryogenesis. Science *360*.

Furlong, E.E.M., and Levine, M. (2018). Developmental enhancers and chromosome topology. Science *361*, 1341–1345.

Ghavi-Helm, Y., Jankowski, A., Meiers, S., Viales, R.R., Korbel, J.O., and Furlong, E.E.M. (2019). Highly rearranged chromosomes reveal uncoupling between genome topology and gene expression. Nat. Genet. *51*, 1272–1282.

Granja, J.M., Corces, M.R., Pierce, S.E., Bagdatli, S.T., Choudhry, H., Chang, H.Y., and Greenleaf, W.J. (2021). ArchR is a scalable software package for integrative single-cell chromatin accessibility analysis. Nat. Genet. *53*, 403–411.

Gratz, S.J., Rubinstein, C.D., Harrison, M.M., Wildonger, J., and O'Connor-Giles, K.M. (2015). CRISPR-Cas9 genome editing in *Drosophila*. Curr. Protoc. Mol. Biol. *111*, 31.2.1–31.2.20.

Gratz, S.J., Ukken, F.P., Rubinstein, C.D., Thiede, G., Donohue, L.K., Cummings, A.M., and O'Connor-Giles, K.M. (2014). Highly specific and efficient CRISPR/Cas9-catalyzed homology-directed repair in *Drosophila*. Genetics *196*, 961–971.

Hanlon, C.D., and Andrew, D.J. (2016). *Drosophila* FoxL1 non-autonomously coordinates organ placement during embryonic development. Dev. Biol. *419*, 273–284.

Huang, Y., McCarthy, D.J., and Stegle, O. (2019). Vireo: Bayesian demultiplexing of pooled single-cell RNA-seq data without genotype reference. Genome Biol *20*, 273.

Jakobsen, J.S., Braun, M., Astorga, J., Gustafson, E.H., Sandmann, T., Karzynski, M., Carlsson, P., and Furlong, E.E.M. (2007). Temporal ChIP-on-chip reveals Biniou as a universal regulator of the visceral muscle transcriptional network. Genes Dev *21*, 2448–2460.

Junion, G., Spivakov, M., Girardot, C., Braun, M., Gustafson, E.H., Birney, E., and Furlong, E.E.M. (2012). A transcription factor collective defines cardiac cell fate and reflects lineage history. Cell *148*, 473–486.

Klapper, R., Stute, C., Schomaker, O., Strasser, T., Janning, W., Renkawitz-Pohl, R., and Holz, A. (2002). The formation of syncytia within the visceral musculature of the *Drosophila* midgut is dependent on duf, sns and mbc. Mech. Dev. *110*, 85–96.

Kögler, A.C., Kherdjemil, Y., Bender, K., Rabinowitz, A., Marco-Ferreres, R., and Furlong, E.E.M. (2021). Extremely rapid and reversible optogenetic perturbation of nuclear proteins in living embryos. Dev. Cell *56*, 2348–2363.e8.

Korsunsky, I., Millard, N., Fan, J., Slowikowski, K., Zhang, F., Wei, K., Baglaenko, Y., Brenner, M., Loh, P.R., and Raychaudhuri, S. (2019). Fast, sensitive and accurate integration of single-cell data with harmony. Nat. Methods *16*, 1289–1296.

Kudron, M.M., Victorsen, A., Gevirtzman, L., Hillier, L.W., Fisher, W.W., Vafeados, D., Kirkey, M., Hammonds, A.S., Gersch, J., Ammouri, H., et al. (2018). The modern resource: genome-wide binding profiles for hundreds of *Drosophila* and *Caenorhabditis elegans* transcription factors. Genetics *208*, 937–949.

Kvon, E.Z., Kazmar, T., Stampfel, G., Yáñez-Cuna, J.O., Pagani, M., Schernhuber, K., Dickson, B.J., and Stark, A. (2014). Genome-scale functional characterization of *Drosophila* developmental enhancers in vivo. Nature *512*, 91–95.

Langmead, B., and Salzberg, S.L. (2012). Fast gapped-read alignment with Bowtie 2. Nat. Methods *9*, 357–359.

Lee, D.M., and Chen, E.H. (2019). *Drosophila* myoblast fusion: invasion and resistance for the ultimate union. Annu. Rev. Genet. *53*, 67–91.

Lee, H.-H., Zaffran, S., and Frasch, M. (2006). Development of the larval visceral musculature. In Muscle Development in *Drosophila*, H. Sink, ed. (Landes Bioscience), pp. 62–78.

Li, H., and Durbin, R. (2009). Fast and accurate short read alignment with Burrows-Wheeler transform. Bioinformatics *25*, 1754–1760.

Li, H., Handsaker, B., Wysoker, A., Fennell, T., Ruan, J., Homer, N., Marth, G., Abecasis, G., Durbin, R., and 1000 Genome Project Data Processing Subgroup. (2009). The sequence alignment/map format and SAMtools. Bioinformatics *25*, 2078–2079.

Lilly, B., Zhao, B., Ranganayakulu, G., Paterson, B.M., Schulz, R.A., and Olson, E.N. (1995). Requirement of MADS domain transcription factor D-MEF2 for muscle formation in *Drosophila*. Science *267*, 688–693.

Lin, S.C., Lin, M.H., Horváth, P., Reddy, K.L., and Storti, R.V. (1997). PDP1, a novel *Drosophila* PAR domain bZIP transcription factor expressed in developing mesoderm, endoderm and ectoderm, is a transcriptional regulator of somatic muscle genes. Development *124*, 4685–4696.

Liu, Y.H., Jakobsen, J.S., Valentin, G., Amarantos, I., Gilmour, D.T., and Furlong, E.E.M. (2009). A systematic analysis of Tinman function reveals Eya and JAK-STAT signaling as essential regulators of muscle development. Dev. Cell *16*, 280–291.

McKenna, A., Hanna, M., Banks, E., Sivachenko, A., Cibulskis, K., Kernytsky, A., Garimella, K., Altshuler, D., Gabriel, S., Daly, M., and DePristo, M.A. (2010). The genome analysis toolkit: a MapReduce framework for analyzing next-generation DNA sequencing data. Genome Res *20*, 1297–1303.

Meiler, A., Marchiano, F., Haering, M., Weitkunat, M., Schnorrer, F., and Habermann, B.H. (2021). AnnoMiner is a new web-tool to integrate epigenetics, transcription factor occupancy and transcriptomics data to predict transcriptional regulators. Sci. Rep. *11*, 15463.

Miller, D.E., Cook, K.R., Arvanitakis, A.V., and Hawley, R.S. (2016). Third chromosome balancer inversions disrupt protein-coding genes and influence distal recombination events in *Drosophila melanogaster*. G3 (Bethesda) *6*, 1959–1967.

Miller, D.E., Cook, K.R., Hemenway, E.A., Fang, V., Miller, A.L., Hales, K.G., and Hawley, R.S. (2018). The molecular and genetic characterization of second chromosome balancers in *Drosophila melanogaster*. G3 (Bethesda) *8*, 1161–1171.

Minnoye, L., Marinov, G.K., Krausgruber, T., Pan, L., Marand, A.P., Secchia, S., Greenleaf, W.J., Furlong, E.E.M., Zhao, K., Schmitz, R.J., et al. (2021). Chromatin accessibility profiling methods. Nat. Rev. Methods Prim. *1*, 1–24.

Özel, M.N., Simon, F., Jafari, S., Holguera, I., Chen, Y.C., Benhra, N., El-Danaf, R.N., Kapuralin, K., Malin, J.A., Konstantinides, N., and Desplan, C. (2021). Neuronal diversity and convergence in a visual system developmental atlas. Nature *589*, 88–95.

Pijuan-Sala, B., Griffiths, J.A., Guibentif, C., Hiscock, T.W., Jawaid, W., Calero-Nieto, F.J., Mulas, C., Ibarra-Soria, X., Tyser, R.C.V., Ho, D.L.L., et al. (2019). A single-cell molecular map of mouse gastrulation and early organogenesis. Nature *566*, 490–495.

Pijuan-Sala, B., Wilson, N.K., Xia, J., Hou, X., Hannah, R.L., Kinston, S., Calero-Nieto, F.J., Poirion, O., Preissl, S., Liu, F., and Göttgens, B. (2020). Single-cell chromatin accessibility maps reveal regulatory programs driving early mouse organogenesis. Nat. Cell Biol. *22*, 487–497.

Plass, M., Solana, J., Alexander Wolf, F., Ayoub, S., Misios, A., Glažar, P., Obermayer, B., Theis, F.J., Kocks, C., and Rajewsky, N. (2018). Cell type atlas and lineage tree of a whole complex animal by single-cell transcriptomics. Science *360*, eaaq1723.

Quinlan, A.R., and Hall, I.M. (2010). BEDTools: a flexible suite of utilities for comparing genomic features. Bioinformatics *26*, 841–842.

Ramírez, F., Ryan, D.P., Grüning, B., Bhardwaj, V., Kilpert, F., Richter, A.S., Heyne, S., Dündar, F., and Manke, T. (2016). deepTools2: a next generation web server for deep-sequencing data analysis. Nucleic Acids Res *44*, W160–W165.

Reddington, J.P., Garfield, D.A., Sigalova, O.M., Karabacak Calviello, A., Marco-Ferreres, R., Girardot, C., Viales, R.R., Degner, J.F., Ohler, U., and Furlong, E.E.M. (2020). Lineage-resolved enhancer and promoter usage during a time course of embryogenesis. Dev. Cell *55*, 648–664.e9.

Reim, I., and Frasch, M. (2010). Genetic and genomic dissection of cardiogenesis in the Drosophila model. Pediatr. Cardiol. *31*, 325–334.

Reiter, F., Wienerroither, S., and Stark, A. (2017). Combinatorial function of transcription factors and cofactors. Curr. Opin. Genet. Dev. *43*, 73–81.

Riechmann, V., Irion, U., Wilson, R., Grosskortenhaus, R., and Leptin, M. (1997). Control of cell fates and segmentation in the *Drosophila* mesoderm. Development *124*, 2915–2922.

Rivera, J., Keränen, S.V.E., Gallo, S.M., and Halfon, M.S. (2019). REDfly: the transcriptional regulatory element database for *Drosophila*. Nucleic Acids Res *47*, D828–D834.

Rousseeuw, P.J. (1987). Silhouettes: a graphical aid to the interpretation and validation of cluster analysis. J. Comput. Appl. Math. *20*, 53–65.

Rudolf, A., Buttgereit, D., Jacobs, M., Wolfstetter, G., Kesper, D., Pütz, M., Berger, S., Renkawitz-Pohl, R., Holz, A., and Önel, S.F. (2014). Distinct genetic programs guide *Drosophila* circular and longitudinal visceral myoblast fusion. BMC Cell Biol *15*, 27.

San Martin, B., and Bate, M. (2001). Hindgut visceral mesoderm requires an ectodermal template for normal development in *Drosophila*. Development *128*, 233–242.

Sandmann, T., Girardot, C., Brehme, M., Tongprasit, W., Stolc, V., and Furlong, E.E.M. (2007a). A core transcriptional network for early mesoderm development in *Drosophila melanogaster*. Genes Dev *21*, 436–449.

Sandmann, T., Jakobsen, J.S., and Furlong, E.E.M. (2007b). ChIP-on-chip protocol for genome-wide analysis of transcription factor binding in *Drosophila melanogaster* embryos. Nat. Protoc. *1*, 2839–2855.

Sandmann, T., Jensen, L.J., Jakobsen, J.S., Karzynski, M.M., Eichenlaub, M.P., Bork, P., and Furlong, E.E.M. (2006). A temporal map of transcription factor activity: Mef2 directly regulates target genes at all stages of muscle development. Dev. Cell *10*, 797–807.

Satija, R., Farrell, J.A., Gennert, D., Schier, A.F., and Regev, A. (2015). Spatial reconstruction of single-cell gene expression data. Nat. Biotechnol. *33*, 495–502.

Satpathy, A.T., Granja, J.M., Yost, K.E., Qi, Y., Meschi, F., McDermott, G.P., Olsen, B.N., Mumbach, M.R., Pierce, S.E., Corces, M.R., et al. (2019). Massively parallel single-cell chromatin landscapes of human immune cell development and intratumoral T cell exhaustion. Nat. Biotechnol. *37*, 925–936.

Schaub, C., and Frasch, M. (2013). Org-1 is required for the diversification of circular visceral muscle founder cells and normal midgut morphogenesis. Dev. Biol. *376*, 245–259.

Schep, A.N., Wu, B., Buenrostro, J.D., and Greenleaf, W.J. (2017). ChromVAR: inferring transcription-factor-associated accessibility from single-cell epigenomic data. Nat. Methods *14*, 975–978.

Schindelin, J., Arganda-Carreras, I., Frise, E., Kaynig, V., Longair, M., Pietzsch, T., Preibisch, S., Rueden, C., Saalfeld, S., Schmid, B., et al. (2012). Fiji: an open-source platform for biological-image analysis. Nat. Methods *9*, 676–682.

Schor, I.E., Bussotti, G., Maleš, M., Forneris, M., Viales, R.R., Enright, A.J., and Furlong, E.E.M. (2018). Non-coding RNA expression, function, and variation during *Drosophila* embryogenesis. Curr. Biol. *28*, 3547–3561.e9.

Spitz, F., and Furlong, E.E.M. (2012). Transcription factors: from enhancer binding to developmental control. Nat. Rev. Genet. *13*, 613–626.

Stuart, T., Srivastava, A., Madad, S., Lareau, C.A., and Satija, R. (2021). Single-cell chromatin state analysis with Signac. Nat. Methods *18*, 1333–1341.

Tao, Y., Wang, J., Tokusumi, T., Gajewski, K., and Schulz, R.A. (2007). Requirement of the LIM homeodomain transcription factor tailup for normal heart and hematopoietic organ formation in *Drosophila melanogaster*. Mol. Cell. Biol. *27*, 3962–3969.

Tomancak, P., Beaton, A., Weiszmann, R., Kwan, E., Shu, S.Q., Lewis, S.E., Richards, S., Ashburner, M., Hartenstein, V., Celniker, S.E., et al. (2002). Systematic determination of patterns of gene expression during *Drosophila* embryogenesis. Genome Biol *3*, RESEARCH0088.

Tyser, R.C.V., Ibarra-Soria, X., McDole, K., Arcot Jayaram, S., Godwin, J., van den Brand, T.A.H., Miranda, A.M.A., Scialdone, A., Keller, P.J., Marioni, J.C., and Srinivas, S. (2021). Characterization of a common progenitor pool of the epicardium and myocardium. Science *371*.

Wagner, D.E., Weinreb, C., Collins, Z.M., Briggs, J.A., Megason, S.G., and Klein, A.M. (2018). Single-cell mapping of gene expression landscapes and lineage in the zebrafish embryo. Science *360*, 981–987.

Yin, Z., and Frasch, M. (1998). Regulation and function of tinman during dorsal mesoderm induction and heart specification in *Drosophila*. Dev. Genet. *22*, 187–200.

Zaffran, S., Küchler, A., Lee, H.H., and Frasch, M. (2001). biniou (FoxF), a central component in a regulatory network controlling visceral mesoderm development and midgut morphogenesis in *Drosophila*. Genes Dev *15*, 2900–2915.

Zhang, Y., Liu, T., Meyer, C.A., Eeckhoute, J., Johnson, D.S., Bernstein, B.E., Nusbaum, C., Myers, R.M., Brown, M., Li, W., and Liu, X.S. (2008). Model-based analysis of ChIP-Seq (MACS). Genome Biol *9*, R137.

Zinzen, R.P., Girardot, C., Gagneur, J., Braun, M., and Furlong, E.E.M. (2009). Combinatorial binding predicts spatio-temporal cis-regulatory activity. Nature *462*, 65–70.

Zmojdzian, M., and Jagla, K. (2013). Tailup plays multiple roles during cardiac outflow assembly in *Drosophila*. Cell Tissue Res *354*, 639–645.

STAR★METHODS

KEY RESOURCES TABLE

REAGENT or RESOURCE	SOURCE	IDENTIFIER
**Antibodies**		
Donkey anti-rabbit IgG-PE conjugate	Biolegend	Cat# 406421; RRID: AB_2563484
Alexa Fluor conjugated secondary antibodies	Thermo Fisher Scientific	Various
Rat anti-Tropomyosin	Babraham	Cat# P6694
Mouse anti-Fasciclin III	DSHB	Cat# 7G10; RRID: AB_528238
Chicken anti-beta-Galactosidase	Abcam	Cat# ab9361; RRID: AB_307210
Rabbit anti-Mef2	Furlong laboratory, EMBL	N/A
Rabbit anti-Biniou	Furlong laboratory, EMBL	N/A
Sheep Anti-Digoxigenin Fab fragments Antibody, POD Conjugated	Roche	Cat# 11207733910; RRID: AB_514500
**Bacterial and virus strains**		
XL1-Blue Competent cells	Custom made. Furlong laboratory, EMBL	N/A
**Chemicals, peptides, and recombinant proteins**		
Prolong Gold antifade reagent with DAPI	Thermo Fisher Scientific	Cat# P36931
Complete Protease Inhibitor Cocktail	Roche	Cat# 11697498001
Bovine Serum Albumin	Sigma	Cat# A9418-500G<br>CAS: 9048-46-8
Triton X-100	Sigma	Cat# T8787<br>CAS: 9036-19-5
Igepal CA630NP-40	Sigma	Cat# I3021<br>CAS: 9002-93-1
DAPI	Sigma	Cat# D9542-1MG<br>CAS: 28718-90-3
TD Tagment DNA Buffer	Illumina	Cat# 15027866
Nextera PCR Master (NPM) Mix	Illumina	Cat# #15027920
Formaldehyde solution about 37% (fixative for sci-ATAC-seq)	Sigma	Cat# 1040031000
Proteinase K	Qiagen	Cat# 19131
Formaldehyde ultra-pure methanol free 16% (fixative for immunostainings and RNA in situ)	Polysciences	Cat #18814-10<br>CAS: 50-00-0
**Critical commercial assays**		
Qubit dsDNA HS Assay Kit	Thermo Fisher Scientific	Cat# Q32854
DNA Clean & Concentrator-5	Zymo	Cat# D4014
DIG RNA Labelling Mixture	Roche	Cat# 11277073910
TSA Plus Cy3 and Fluor kit	Perkin Elmer	Cat# NEL753001KT
AMPureXP beads	Beckman Coulter	Cat# A63881
**Deposited data**		
BDGP gene expression data	Tomancak et al. (2002)	https://insitu.fruitfly.org/insitu-mysql-dump/insitu_annot.csv.gz
Mef2 -/- gene expression data	Sandmann et al. (2006)	ArrayExpress: E-TABM-57 and http://furlonglab.embl.de/labData/publications/before_2009/SandmannT-et-al-2006_DevCell/data/mef2_mutant_expression_data.zip
sci-ATAC-seq sequence data	This study	ArrayExpress: E-MTAB-9034
modERN ChIP-seq datasets	Kudron et al. (2018)	https://epic.gs.washington.edu/modERN/

(Continued on next page)

Continued

REAGENT or RESOURCE	SOURCE	IDENTIFIER
Mesoderm/muscle TF datasets	Cunha et al. (2010); Jakobsen et al. (2007); Junion et al. (2012); Zinzen et al. (2009)	All ChIP data are available from ArrayExpress (accession numbers E-TABM-648, E-TABM-649, E-TABM-650, E-TABM-651, E-TABM-652 and E-TABM-1184). The CRMs and ChIP peak coordinates are available at http://furlonglab.embl.de/data
DNase I Hypersensitive Sites	Reddington et al. (2020)	ArrayExpress: E-MTAB-8881
Enhancer activity database (CAD4)	Cusanovich et al. (2018a)	Table S13
Wild-type *Drosophila* melanogaster whole-embryo sci-ATAC-seq peakset	Cusanovich et al. (2018a)	Table S1
Experimental models: Organisms/Strains		
D.melanogaster wild-type (canton S)	Furlong laboratory, EMBL	N/A
D.melanogaster: w[1118]; PBac{y[+mDint2]=vas-Cas9}VK00027	Bloomington Drosophila Stock Center	RRID:BDSC_51324
Oligonucleotides		
CRISPR gRNAs and ssODNs	See Table S4	N/A
Recombinant DNA		
Plasmid: pU6-BbsI-chiRNA	Addgene	ID 45946
Plasmid: pU6-Mef2-gRNA	This study	Lab stocks ID: 2768
Plasmid: pU6-tin-gRNA	This study	Lab stocks ID: 2769
Plasmid: pU6-bap-gRNA	This study	Lab stocks ID: 2770
Plasmid: pU6-bin-gRNA	This study	Lab stocks ID: 2771
Plasmid: pOT2-luna. BDGP EST clone HL07808	DGRC	Cat# 12791
Plasmid: pOTB7-Nk7.1. BDGP EST clone AT09939	DGRC	Cat# 1025239
Plasmid: pOT2-CG14655. BDGP EST clone GH23506	DGRC	Cat# 4046
Software and algorithms		
SAMtools	Li et al. (2009)	http://www.htslib.org/ RRID:SCR_002105
BEDTools	Quinlan and Hall (2010)	http://bedtools.readthedocs.io/en/latest/ RRID:SCR_006646
deepTools	Ramírez et al. (2016)	https://deeptools.readthedocs.io/en/develop/ RRID:SCR_016366
MACS2	Zhang et al. (2008)	https://doi.org/10.5281/zenodo.3748809 RRID:SCR_013291
cisTopic	Bravo González-Blas et al. (2019)	https://github.com/aertslab/cisTopic
Seurat	Satija et al. (2015)	https://satijalab.org/seurat/ RRID:SCR_016341
flyCRISPR target finder	Gratz et al. (2014)	http://targetfinder.flycrispr.neuro.brown.edu/
chromVAR	Schep et al. (2017)	https://doi.org/10.18129/B9.bioc.chromVAR
Signac	Stuart et al. (2021)	https://satijalab.org/signac/
Trajectory and pseudotime inference	Satpathy et al. (2019)	https://github.com/GreenleafLab/10x-scATAC-2019
ArchR	Granja et al. (2021)	https://www.archrproject.com/
GATK	McKenna et al. (2010)	https://gatk.broadinstitute.org/hc/en-us RRID:SCR_001876

(*Continued on next page*)

Continued		
REAGENT or RESOURCE	SOURCE	IDENTIFIER
Trim Galore	The Babraham Institute	https://doi.org/10.5281/zenodo.5127899
BWA	Li and Durbin (2009)	http://bio-bwa.sourceforge.net/ RRID:SCR_010910
Picard	Broad Institute	https://broadinstitute.github.io/picard/ RRID:SCR_006525
Vireo	Huang et al. (2019)	https://vireosnp.readthedocs.io/en/latest/
Harmony	Korsunsky et al. (2019)	https://portals.broadinstitute.org/harmony/
R	The R foundation	https://www.r-project.org/
Fiji	Schindelin et al. (2012)	https://fiji.sc/
sci-ATAC-seq processing pipeline	Cusanovich et al. (2018a)	https://atlas.gs.washington.edu/fly-atac/
Trimmomatic	Bolger et al. (2014)	http://www.usadellab.org/cms/?page=trimmomatic RRID:SCR_011848
Bowtie2	Langmead and Salzberg (2012)	http://bowtie-bio.sourceforge.net/bowtie2/index.shtml RRID:SCR_016368
Banding score calculation	Cusanovich et al. (2018b)	https://atlas.gs.washington.edu/mouse-atac/
Other		
Illumina NextSeq 500 High Output	Illumina	N/A
BD FACSMelody Cell Sorter	BD Biosciences	N/A
Zeiss LSM780	Zeiss	N/A
Plan Apochromat 20x/0.8 objective	Zeiss	N/A

RESOURCE AVAILABILITY

Lead contact

Further information and requests for resources/reagents should be directed to Eileen Furlong (furlong@embl.de) who will coordinate their provision.

Materials availability

The fly lines used in this study were generated by the authors as described in the methods section and are maintained for the community by the Furlong lab at EMBL. Only costs to cover post and packaging will be requested. Non-commercial antibodies used in this study were published previously.

Data and code availability

- All raw sequencing data generated in this study has been submitted to the EMBL-EBI ArrayExpress database (https://www.ebi.ac.uk/arrayexpress/) and are available under accession number E-MTAB-10489. Processed data, including count tables and the VCF files used for genotyping, can be downloaded from http://furlonglab.embl.de/data/. Clustered data can be interactively explored and downloaded at http://furlonglab.embl.de/ss/Drosophila-Mesoderm-Chromatin-Accessibility.
- This paper does not report original code.
- Any additional information required to reanalyze the data reported in this paper is available from the lead contact upon reasonable request.

EXPERIMENTAL MODEL AND SUBJECT DETAILS

D. melanogaster model

All fly lines were raised on standard food between 18°C and 25°C. Embryo collections were performed on apple juice agar plates with yeast paste at 25°C and 60% humidity. For wild-type experiments, embryos were collected from Canton S flies. For CRISPR knock-out experiments, we made use of an isogenic fly line that expresses Cas9 in the germline under the Vasa promoter w[1118]; PBac{y[+mDint2]=vas-Cas9}VK00027 (Bloomington stock 51324). This line is marked with a 3xP3-GFP reporter that is expressed in the brain and adult eyes.

CellPress
OPEN ACCESS

Generation of CRISPR strains

Balancer chromosomes are highly inverted chromosomes that prevent the recovery of recombinants by suppressing genetic recombination between homologous chromosomes during meiosis. They are typically homozygous lethal, and maintained in *trans* to a non-balancer homologous chromosome. When placed in *trans* to a recessive lethal mutation, the only embryos that can survive to adulthood are the trans-heterozygous mutation/balancer, with the homozygous mutation/mutation and balancer/balancer offspring being lethal. Recessive lethal mutations can thereby be maintained in *trans* to a balancer for decades (indefinitely). However, as any additional spurious mutations on mutant of interest's chromosome also cannot recombine off the chromosome due to the presence of the balancer chromosome, old mutant stocks naturally accumulate other deleterious mutations.

As the loss-of-function lines for all four TFs assessed in this study were generated twenty or more years ago, they will have accumulated many additional mutations, which are also maintained by the balancer chromosomes. In addition, as they were made by different labs at different stages, they also have different genetic backgrounds. We therefore initiated this study by generating clean loss-of-function mutants for all four TFs in the same isogenic background. As the previous alleles were molecularly characterized and demonstrated to be loss-of-function, we used CRISPR induced template directed homology to regenerate the same loss-of-function alleles for each factor. Specifically, we regenerated the *Mef2* 22.21 allele (Flybase ID FBal0033789; (Bour et al., 1995)), *tinman* EC40 allele (Flybase ID FBal0032861; (Bodmer, 1993)) and *biniou* R22 allele (Flybase ID FBal0043738; (Zaffran et al., 2001)). These are all single-nucleotide nonsense mutations that introduce a premature stop codon and are therefore protein nulls. As the gene *bagpipe* (Flybase ID FBgn0004862) does not have a characterized loss-of-function allele, the mutant phenotype was characterized using a deficiency, we applied the same CRISPR approach to introduce a nonsense mutation at 3R:G21389189T, which is located in the first exon and 180 bp before the TF's DNA binding domain. These mutations were introduced in a clean, isogenic and fully sequenced fly line that expresses Cas9 in the germline under the Vasa promoter w[1118]; PBac{y[+mDint2]=vas-Cas9}VK00027 (Bloomington stock 51324).

A single stranded oligonucleotide (ssODN) was designed for each locus (Table S4) to serve as a template for HDR following the Cas9 induced double strand break, based on the protocol available on the flyCRISPR website (https://flycrispr.org/protocols/ssodn/) (Gratz et al., 2015). The template ssODNs were designed to include additional features besides the intended single-nucleotide nonsense mutation (Figure S3A): (1) a restriction site for SacI (GAGCTC; NEB) was introduced downstream the premature stop codon to be used for screening. (2) A thymine nucleotide was inserted immediately upstream the SacI restriction site, causing a frame-shift mutation that generates a second premature stop codon (TGA); this would serve to terminate translation in case of read-through after the first stop codon. (3) point mutations in the PAM or the gRNA seed to prevent re-cutting by Cas9. The ssODNs were synthesized by Integrated DNA Technologies (Coralville, IA, United States of America) (IDT). A single gRNA against each target locus (Table S4) was designed using flyCRISPR target finder (http://targetfinder.flycrispr.neuro.brown.edu/) (Gratz et al., 2014). The gRNAs were cloned in the vector pBs-U6-gRNA-BbsI (Addgene #45946) and injected together with the ssODNs into embryos of the Vasa-Cas9 fly line described above.

Emerging flies were crossed back to the same isogenic Vasa-Cas9 line in trans to a sequenced balancer for chromosome 2 (If/Cyo, IsoVasCas9; Mef2 allele) or chromosome 3 (IsoVasCas9, Sb/TM3 Ser; other alleles). Screening was performed by SacI restriction digestion of the PCR amplified locus. We confirmed that the correct mutant alleles were regenerated for each locus by three independent methods; (1) the intended nonsense mutations were confirmed by Sanger sequencing, (2) by a genetic complementation test, which showed that the new alleles non- complement the lines carrying the "original" mutant alleles described above, as expected, and (3) immunostaining showed that the new alleles recapitulate the known mutant phenotypes (Figure S3B).

METHOD DETAILS

Embryo fixation and nuclear isolation

Drosophila melanogaster embryos were collected and fixed as previously described (Bonn et al., 2012b, 2012a; Sandmann et al., 2007b). In summary, embryos were collected in staged two-hour windows following three one-hour pre-lays to clear the females and synchronize the collections, which were aged at 25°C to the corresponding time window (3-5 hr, 4-6 hr, 5-7 hr, 6-8 hr, 7-9 hr, 8-10 hr, 9-11 hr and 10-12 hr for wild-type (Canton S) collections, 10-12 hr for *Mef2*, 5-7 hr for *tinman* and 6-8 hr for *biniou* and *bagpipe* mutant embryo collections). Embryos were dechorionated in 50% bleach for 2 min and fixed with 1.8% formaldehyde for 15 min. For *Mef2* hand-sorted mutant embryo collections, the *Mef2* mutant allele was placed in trans to a GFP marked balancer chromosome (CyO, twi-Gal4, UAS-GFP) and the homozygous mutant embryos (GFP negative) were hand-sorted from their siblings under a dissection microscope prior to fixation. After 15 minutes of formaldehyde fixation, embryos were quenched with glycine, washed, dried, snap frozen in liquid nitrogen and stored at − 80 °C. Embryo dissociation and nuclear isolation were performed using a dounce homogenizer and a 22G needle as previously described (Bonn et al., 2012b). Nuclei were resuspended in nuclear freezing buffer (50 mM Tris at pH 8.0, 25% glycerol, 5 mM Mg(OAc)2, 0.1 mM EDTA, 5 mM DTT, 1× protease inhibitor cocktail (Roche #11697498001)), snap frozen in liquid nitrogen and stored at -80 °C.

Nuclear staining for FANS of mesoderm / muscle populations

One day prior to the sci-ATAC-seq experiments, aliquots of 10 million nuclei obtained from wild-type, *bap* and *bin* collections were prepared for Fluorescence-Activated Nuclear Sorting (FANS) using an improved BiTS protocol as described previously (Reddington

et al., 2020). Primary antibody staining was performed overnight at 4 °C in 400 μL 1X PBS supplemented with 5% BSA, 0.1% TritonX-100 and 1× protease inhibitor cocktail (Roche #11697498001). Rabbit primary antibodies anti-Mef2 and anti-Biniou (both from (Reddington et al., 2020); 1:1000 dilution) were used to mark all mesoderm or myogenic mesoderm depending on stage and to mark visceral muscle (VM) primordia respectively. Secondary antibody staining was performed by incubation with fluorescently labelled donkey anti-rabbit IgG-PE conjugate (Biolegend #406421; 1:200 dilution) for 1 hour at 4 °C in 400 μl 1X PBS supplemented with 5% BSA, 0.1% TritonX-100 and 1× protease inhibitor cocktail (Roche #11697498001).

Generation of sci-ATAC-seq libraries

Generation of sci-ATAC-seq libraries was performed largely as previously described (Cusanovich et al., 2018a), with some modifications. Nuclei were washed twice by pelleting and resuspending in 1 mL 1X PBS supplemented with 0.1% TritonX-100 and 1× protease inhibitor cocktail (Roche #11697498001). Nuclei were stained with 3 μM DAPI and 2,500 DAPI+ (and Mef2+ or Biniou+ for mesoderm / visceral muscle sorting) nuclei were sorted into each well of a 96-well plate containing 5 μL of Omni-ATAC buffer (Corces et al., 2017) supplemented with 1× protease inhibitor cocktail (Roche #11697498001) and 12 μL of TD buffer (Illumina #15027866) in each well. Tagmentation was performed by adding 2 μl of each of the 96 custom and uniquely indexed Tn5 transposomes (2.5 uM; provided by Illumina as part of a collaborative agreement) and incubating at 55 °C for 2 hours. Following the second sorting step and reverse-crosslinking, tagmented DNA was PCR amplified by adding 5 μL of 5 μM forward and reverse indexed primers (Cusanovich et al., 2018a), 7.5 μl of NPM polymerase master mix (Illumina #15027920) and BSA (2X final concentration) to each well and by running the following cycling conditions: 72 °C 5 min, 98 °C 30 s; 98 °C 10 s, 63 °C 30 s, 19–20 cycles; 72 °C 1 min, hold at 10 °C. The optimal number of cycles for each library was determined beforehand by monitoring amplification on a qPCR machine for a set of test wells. Libraries were sequenced on an Illumina NextSeq 500 sequencer High Capacity 150 PE kit (Illumina) using custom primers (sequencing primers for read 1-2 and for index 1-2) and a custom sequencing recipe previously described (Amini et al., 2014).

Immunostaining and *in situ* hybridization

Whole-mount *Drosophila* embryo immunostaining and fluorescent *in situ* hybridization (FISH) were performed as previously described (Schor et al., 2018). Fixation of overnight embryo collections was carried out in 4% formaldehyde (from a 16% formaldehyde ultra-pure methanol free stock (Polysciences #18814-10)) for 20 minutes. Immunostaining was performed with the following primary antibodies and dilutions: rat anti-Tropomyosin (1:4000) (Babraham #P6694), mouse anti-Fasciclin III (1:500) (DSHB #7G10), chicken anti-beta-Galactosidase (1:500) (Abcam #ab9361), rabbit anti-Mef2 (1:200) (Furlong lab) and rabbit anti-Biniou (1:200) (Furlong lab). Alexa Fluor conjugates (Thermo Fisher Scientific) were used as secondary antibodies (1:500). Digoxigenin-labeled RNA *in situ* probes for *luna*, *Nk7.1*, and *CG14655* were prepared from corresponding EST clones using DIG RNA Labelling Mixture (Roche #11277073910) and the fluorescent detection of mRNA expression was performed using a Tyramide Signal Amplification (TSA) kit (Perkin Elmer #NEL753001KT). Stained embryos were mounted in ProLong Gold Antifade reagent (Thermo Fisher Scientific # P36931) and imaged with a Zeiss LSM780 confocal microscope using a Plan Apochromat 20x/0.8 objective. Images were then visualized in Fiji (Schindelin et al., 2012).

QUANTIFICATION AND STATISTICAL ANALYSIS

Raw data processing and cell assignment

Processing of raw sequencing data was performed with the pipeline provided in (Cusanovich et al., 2018a). In brief, read barcodes presenting sequencing or PCR amplification errors were first corrected to their presumptive match (Levenshtein distance < 3 and distance to next best match > 2); all other barcodes were classified as ambiguous or unknown. Reads were trimmed with Trimmomatic v0.32 (Bolger et al., 2014) and mapped to the dm6 reference genome using Bowtie2 v2.3.4.1 (Langmead and Salzberg, 2012) (with options -X 2000 -3 1).

After removal of PCR duplicates, we proceeded to identify cells and exclude low quality cells by applying three stringent filters (Figure S1A): (1) barcodes were classified as genuine cells if there was no more than 5% uncertainty that they belonged to the higher read depth component of a Gaussian mixture model fitted to the distribution of read counts per barcode (as in (Cusanovich et al., 2018a), QC step 1). (2) As the insert size distribution was evident even in single cells (Figure S1A), we quantified nucleosomal banding per-cell using a fast Fourier transform-based metric with the scripts provided in (Cusanovich et al., 2018b) and retained cells with clear nucleosomal banding (QC step 2). (3) We realized that cells missing a clear sub-nucleosomal peak can still receive a good nucleosomal banding score based on this metric alone (Figure S1A) and therefore applied a third filter to remove these cells (QC step 3), based on per-cell quantification of sub-nucleosomal fragments.

Peak calling and generation of bigwig tracks

Deconvoluted BAM files were generated for each described sample / condition with the script ‘sc_atac_library_deconvoluter.py’ from (Cusanovich et al., 2018a) and were then converted to bigwig files with deepTools ‘bamCoverage’ v2.5.1 (Ramírez et al., 2016) (normalization option: –normalizeUsingRPKM). Deconvoluted BAM files were also used for peak calling, which in all instances was performed with MACS2 v2.2.7.1 (Zhang et al., 2008), using the ‘macs2 callpeak’ command with parameters: ‘–nomodel –keep-dup all –extsize 200 –shift -100’. In all instances, peak summits were first resized to 300 bp and then merged with BEDTools v2.27.1

(Quinlan and Hall, 2010) using the default options. With this approach, any overlapping resized summit is merged to generate master lists of peaks for each dataset, as described in the following sections.

LDA, clustering and UMAP visualization

Accessibility matrices of peaks (rows) by cells (columns) for each described sample / condition were generated with script ‘sc_atac_window_counter.py’ from (Cusanovich et al., 2018a) and subsequently used as input for Latent Dirichlet Allocation (LDA) with cisTopic function ‘runModels’ (v0.2.2, R package) (Bravo González-Blas et al., 2019). Sex chromosomes were removed prior to LDA to avoid sex bias in the clustering, as recommended in (Cusanovich et al., 2018a). The resulting topic by cell matrix was fed into Seurat (v3.2.2, R package) (Satija et al., 2015) as dimensionality reduction for computing UMAP plots (function ‘RunUMAP’) and clustering (function ‘FindClusters’).

Clustering of the wild-type time course

After cell filtering based on the QC metrics described above, the two sci-ATAC-seq replicates were merged giving a combined data set of 24,032 wild-type cells. To maximise the resolution of the mesoderm/muscle chromatin accessibility landscape over the whole time course, we performed two rounds of clustering (Figure S2A; Table S1): first each time point was clustered individually with Seurat v3.2.2 function ‘FindClusters’ based on accessibility quantified on a merged set of 42,076 peaks that were called separately for each time point. This process allowed the identification of 61 Seurat clusters, from which we excluded low quality and suspected collision clusters (12 clusters comprising 2,725 cells) based on the read depth and sex ratio metrics, as described in (Cusanovich et al., 2018a). Accessibility was quantified again at 50,261 merged peaks identified separately for each cluster and the resulting count matrix was used for clustering the full time course. Batch correction was not necessary, as we did not observe a clustering bias for the two replicates (Figure S2B).

Calculation of gene activity scores

Gene activity scores were calculated with script ‘sc_atac_window_counter.py’ from (Cusanovich et al., 2018a) by computing accessibility over the whole gene body plus 500 bp upstream, for genes in the R Bionconductor package ‘TxDb.Dmelanogaster.UCSC.dm6.ensGene_3.4.4’. The resulting gene by cell accessibility matrices were imported in Seurat for downstream analysis.

Cell type annotation

ATAC-seq peaks and genes were tested for differential accessibility in each cluster by logistic regression using Seurat v3.2.2 function ‘FindAllMarkers’, with the total counts per cell given as a latent variable. Features with a positive log fold-change and a Bonferroni adjusted P-value below 0.05 were considered to be markers of a given cluster, while the non-significant features were retained as background. The cluster markers and the background features were matched to activity terms of characterized *in vivo* enhancer activity (Bonn et al., 2012a; Kvon et al., 2014; Rivera et al., 2019) or BDGP gene expression (Tomancak et al., 2002), and each activity term in a given cluster was tested for over-representation against the background with a Fisher’s one-tailed test. As many of the activity terms are highly overlapping, the Fisher’s test p-values were not formally corrected for multiple comparison, and instead to assign cell types we focused on large and consistent enrichments of similar activity terms.

Transcription factor deviation scores

To investigate the relationship between accessibility changes and transcription factor occupancy, we retrieved 16 ChIP datasets of mesodermal factors from our lab (Cunha et al., 2010; Jakobsen et al., 2007; Junion et al., 2012; Zinzen et al., 2009) (Figures 2A and 2B) and 280 ChIP datasets on diverse factors from the modERN database (Kudron et al., 2018) (Figure 2C). In all cases, the peak sets already defined by the authors were used. For analysis with the modERN data, we followed our previously reported strategy (Reddington et al., 2020) to exclude ATAC-seq peaks occupied by any TF with ubiquitous expression, which resulted in 30,318 peaks being retained for analysis from our original list of 50,261 peaks. Deviations in accessibility were calculated with chromVAR v1.10.0 (Schep et al., 2017) and averaged per cell type in Figure 2A and per cell type and time point in Figure 2C.

Pseudotime analysis

To order cells in pseudotime, we identified trajectories for the myogenic lineages and aligned single cells along the trajectories following the approach outlined in (Satpathy et al., 2019). We used the function ‘alignTrajectory’ provided in (Satpathy et al., 2019) to construct trajectories in the UMAP subspace for the somatic (clusters 5, 0, 2, 3, 6), cardiac (clusters 5, 0, 15) and visceral lineages (clusters 5, 13, 8, 7) and to calculate pseudotime along the aligned cells (Table S3). We used functions ‘getTrajectory’ and ‘plotTrajectoryHeatmap’ from ArchR v1.0 (Granja et al., 2021) to reconstruct feature trends across pseudotime and plot the peaks and genes heatmaps in Figure 3B. The binarized accessibility matrix was used as input for peaks and the log-normalized gene activity matrix for genes. For heatmap visualization, we selected the top 10% and 20% most variable peaks and genes across pseudotime.

Identification of DA peaks and genes for muscle subpopulations

Logistic regression was performed to identify differentially accessible (DA) ATAC peaks and genes among muscle subpopulations using Seurat v3.2.2 function ‘FindAllMarkers’ (slot = counts, test.use = LR, logfc.threshold = 0, min.pct = 0.1, latent.vars = total counts

in features per cell). Features with Bonferroni adjusted p-value < 0.001 and log_2 fold-change > 0.5 were considered differentially accessible (Table S2). The log_2 fold-change was calculated using counts matrices scaled by the total counts in features per cell, in order to correct for potential coverage differences among clusters, and using a pseudocount of 10^{-6}. To generate the heatmaps in Figure 2D, the scaled accessibility was averaged per cluster with Seurat function 'AverageExpression'.

Single-nucleus genotyping

A set of discriminatory variants, separating the Vasa-Cas9 and the Balancer chromosomes, was used following the GATK best practice pipeline. Since the balancer chromosomes are homozygous lethal, we analyzed an F1 cross between the balancer and the virginizer line together with the homozygous virginizer line to retrieve the Balancer variants (data from (Ghavi-Helm et al., 2019)). Genomic DNA from more than 30 adult flies from the Vasa-Cas9 line was extracted and prepared for whole-genome sequencing, following the protocol in (Ghavi-Helm et al., 2019).

We performed a joint genotype call with GATK version 4.1.0 (McKenna et al., 2010) using the gDNA reads of the virginizer, virginizer-balancer cross and the Vasa-Cas9 lines. Reads were trimmed with Trim Galore version 0.5.0 (options -q 30 –phred33 –illumina –length 75 –paired) and mapped to the reference dm6 genome with bwa mem (Li and Durbin, 2009) version 0.7.17 (options -T 20). Duplicate reads, unmapped reads and reads not in proper pairs were removed using picard-tools version 1.139 and samtools (Li et al., 2009) version 1.9 (options: -F 1548 -f3). In addition, base quality scores were recalibrated using GATK BaseRecalibrator and ApplyBQSR. Variants were then called with GATK HaplotypeCaller (-G StandardAnnotation –min-base-quality-score=20). The resulting variants were hard filtered with bcftools version 1.9 using two sets of cutoffs leading to a lenient and a stringent set of variants. Filters for stringent set: MQ > 58, MQRankSum > -2,5; MQRankSum < 2,5; QD > 20; SOR < 1.5; FS < 10; ReadPosRankSum > -4; ReadPosRankSum < 4. Filters for lenient set: MQ > 40; MQRankSum > -12,5; MQRankSum < 12,5; QD > 2; SOR < 3; FS < 60; ReadPosRankSum > -8; ReadPosRankSum < 8.

Balancer chromosome alleles were inferred from heterozygous variants in the cross between virginizer and balancer lines. Finally, we excluded contigs other than chromosomes 2, 3 and X, obtaining a total of 104,913 single nucleotide variants (SNVs) separating the Vasa-Cas9 and balancer chromosomes when applying the stringent filters and 465,110 when applying the lenient filters.

Genotypes were demultiplexed using Vireo v0.5.6 (Huang et al., 2019) based on the lenient set of discriminatory SNVs identified between the balancer chromosomes and the common isogenic Vasa-Cas9 genetic background of each mutant. First, cellSNP (https://github.com/single-cell-genetics/cellSNP) was used to pileup aligned reads at each variant for every cell, filtering variants with less than 50 reads or a minor allele frequency of less than 10% (options –minMAF 0.1 –minCOUNT 50 –UMItag=None). This resulted in a set of 201,349 variants for the *Mef2* mutant (chromosome 2 balancer) and between 86,912 and 149,914 variants for *tin*, *bin* and *bap* mutants (chromosome 3 balancer) (Figure S4). Second, allele-specific counts were passed to Vireo in order to assign nuclei to one of the three genotypes using the known reference genomes. Doublet detection was turned off (option –noDoublet) as Cas9/Balancer cells are indistinguishable from barcode collisions between Cas9 and Balancer cells. The barcodes that passed the QC steps described above were further filtered for having been assigned a genotype, while unassigned nuclei were excluded from downstream analysis.

Clustering of the *Mef2* mutant dataset

After cell QC and genotype assignment (Table S5), chromatin accessibility for *Mef2* nuclei was quantified at the 53,133 peaks previously identified in the whole-embryo sci-ATAC-seq dataset (Cusanovich et al., 2018a) and the mesoderm / muscle cells were identified by clustering as described above (5 clusters comprising 2,567 cells). Peaks were called as described above for each mesoderm / muscle cluster and merged with peaks called on each genotype, resulting in 54,609 merged peaks. This peak set was used to quantify accessibility in the 2,567 muscle cells and 739 additional muscle cells that were identified by clustering the *Mef2* hand-sorted sample, totally 3,306 muscle cells. We performed one round of clustering followed by cell type annotation. In the final clustering displayed in Figure 4D, we removed an unrelated neuronal population (78 cells) and a group of 216 interspersed cells that showed poor clustering based on silhouette analysis (Rousseeuw, 1987), a common method to evaluate cluster cohesion and separation (all clusters had an average silhouette width above zero (mean = 0.13) except the removed cells, which had a negative average silhouette width (mean = -0.09), as calculated with R function 'silhouette' from package 'cluster' v2.1.2). For co-clustering with the wild-type time course in Figure 4I (Table S5), chromatin accessibility was quantified at 66,105 peaks obtained from merging wild-type peaks (50,261) with the lists of *Mef2* genotype-cluster peaks defined above. Topic modelling was applied to the count matrix with cisTopic v0.2.2 (Bravo González-Blas et al., 2019) and the topic by cell matrix was batch corrected with Harmony v1.0 (Korsunsky et al., 2019) (theta = 0; wild-type cells were provided as reference with option 'reference_values') prior to UMAP visualization.

Clustering of *tinman*, *bagpipe* and *biniou* mutant datasets

After cell quality control and genotype assignment (Table S5), chromatin accessibility for *bap* and *bin* mutant cells was initially quantified at the 50,261 peaks previously defined for the wild-type time course, and the resulting count matrices were clustered. Peaks were then called as described above for each cell cluster and merged with peaks called on each genotype, resulting in 57,878 and 55,490 merged peaks for *bap* and *bin* respectively. Similar to *Mef2* processing, chromatin accessibility for *tin* was quantified at 53,133 peaks previously identified in a whole-embryo sci-ATAC-seq dataset (Cusanovich et al., 2018a) and the mesoderm / muscle cells were identified by clustering (6 clusters comprising 6,786 cells). Peaks were called for each mesoderm / muscle cluster and merged with peaks called on each genotype, resulting in 60,104 merged peaks. For co-clustering with the wild-type time course

in Figure 5B (Table S5), chromatin accessibility was quantified at 63,842 peaks obtained from merging wild-type peaks (50,261) with the lists of genotype-cluster peaks from each mutant of the three mutant datasets (*tin*, *bap*, *bin*). Topic modelling was applied to the count matrix using cisTopic v0.2.2 (Bravo González-Blas et al., 2019) and the topic-by-cell matrix was batch corrected with Harmony v1.0 (Korsunsky et al., 2019) (theta = 0; each dataset was specified as one batch (wild-type, *tin*, *bap*, *bin*); wild-type cells were provided as reference with option 'reference_values') prior to UMAP visualization and clustering. Cell type labels were very homogenous within each cluster, with an average of 80% of cells having the same label. We therefore assigned the most frequent label as the cell type annotation for a given cluster. Significant imbalances in the proportion of homozygous mutant nuclei obtained in each cluster were identified using a Fisher's exact test against the observed overall proportion (Expected: 25%; Observed: 15%, 19%, 17% for *tin*, *bap* and *bin* respectively).

Differential ATAC peak analysis in *Mef2* mutant embryos

Logistic regression was performed to identify differentially accessible (DA) sites in Mutant1 cluster (1,004 cells) against the somatic cluster (844 cells) using Seurat v3.2.2 function 'FindMarkers' (test.use = LR, logfc.threshold = 0, min.pct = 0.1, latent.vars = total counts in peaks per cell). Out of 8,725 sites tested, 408 had significant differential accessibility (Bonferroni adjusted p-value < 0.05, log_2 fold-change > +/- 0.5, Table S6). To correct for potential coverage differences among clusters, the counts were scaled by the total counts in peaks per cell, prior to the log_2 fold-change calculation. The analysis shown in Figure 6B was performed by testing for differential accessibility between mutant (*Mef2* -/-) and non-mutant cells (*Mef2* +/+ and +/-) across the *Mef2* whole-embryo data (Figure S5B; 3,306 mutant and 9,620 non-mutant cells) or the muscle population (Figure S5B, Muscle cluster; 929 mutant and 1,638 non-mutant cells) respectively. In both cases, differentially accessible sites were identified using logistic regression as described above, keeping the same parameters (logfc.threshold = 0, min.pct = 0.1, latent.vars = total counts in peaks per cell) and significance threshold (Bonferroni adjusted p-value < 0.05, log_2 fold-change > +/- 0.5). To generate the heatmaps in Figure 6C, accessibility was averaged per cluster with Seurat function 'AverageExpression'. Sites residing within 1 kb (+/- 500 bp) centered on a gene TSS were defined as gene-proximal, sites outside this region were considered gene-distal, and putative enhancers. BEDTools v2.27.1 (Quinlan and Hall, 2010) was used to find overlaps between sites and several genomic features (Table S6), including a large collection of characterized embryonic enhancers in transgenic embryos (Bonn et al., 2012a; Kvon et al., 2014; Rivera et al., 2019), occupancy of 10 mesoderm/muscle transcription factors profiled in our lab (Cunha et al., 2010; Jakobsen et al., 2007; Junion et al., 2012; Zinzen et al., 2009), DNase I Hypersensitive Sites (DHSs) of FACS purified mesodermal/muscle cells (Reddington et al., 2020), occupancy of 280 transcription factors from the modERN collection (Kudron et al., 2018) and BDGP gene expression data from in-situ hybridization (Tomancak et al., 2002). In all cases, the peak sets already defined by the authors were used. Sites overlapping a region occupied by Mef2 at 10-12 hr or earlier were classified as Mef2-bound and the non-overlapping sites as unbound. Mef2 motifs were obtained from the collection in (Cannavò et al., 2017) and their presence in sites was scored with function 'AddMotifs' from Signac (Stuart et al., 2021) v1.1.0 (R package). Deciles in Figures 6F and 6I were calculated with function 'ntile' from dplyr v1.0.2 (R package). Gene expression data over a time course of embryogenesis in *Mef2* -/- embryos was obtained from (Sandmann et al., 2006). Genes were considered to be associated with Mef2 if a Mef2-bound ATAC-seq peak resided within their gene body or 5kb upstream of their TSS. By this metric, 1,705 genes were associated with at least one Mef2-bound open chromatin region (Table S6).

Developmental Cell, Volume *57*

Supplemental information

Simultaneous cellular and molecular phenotyping of embryonic mutants using single-cell regulatory trajectories

Stefano Secchia, Mattia Forneris, Tobias Heinen, Oliver Stegle, and Eileen E.M. Furlong

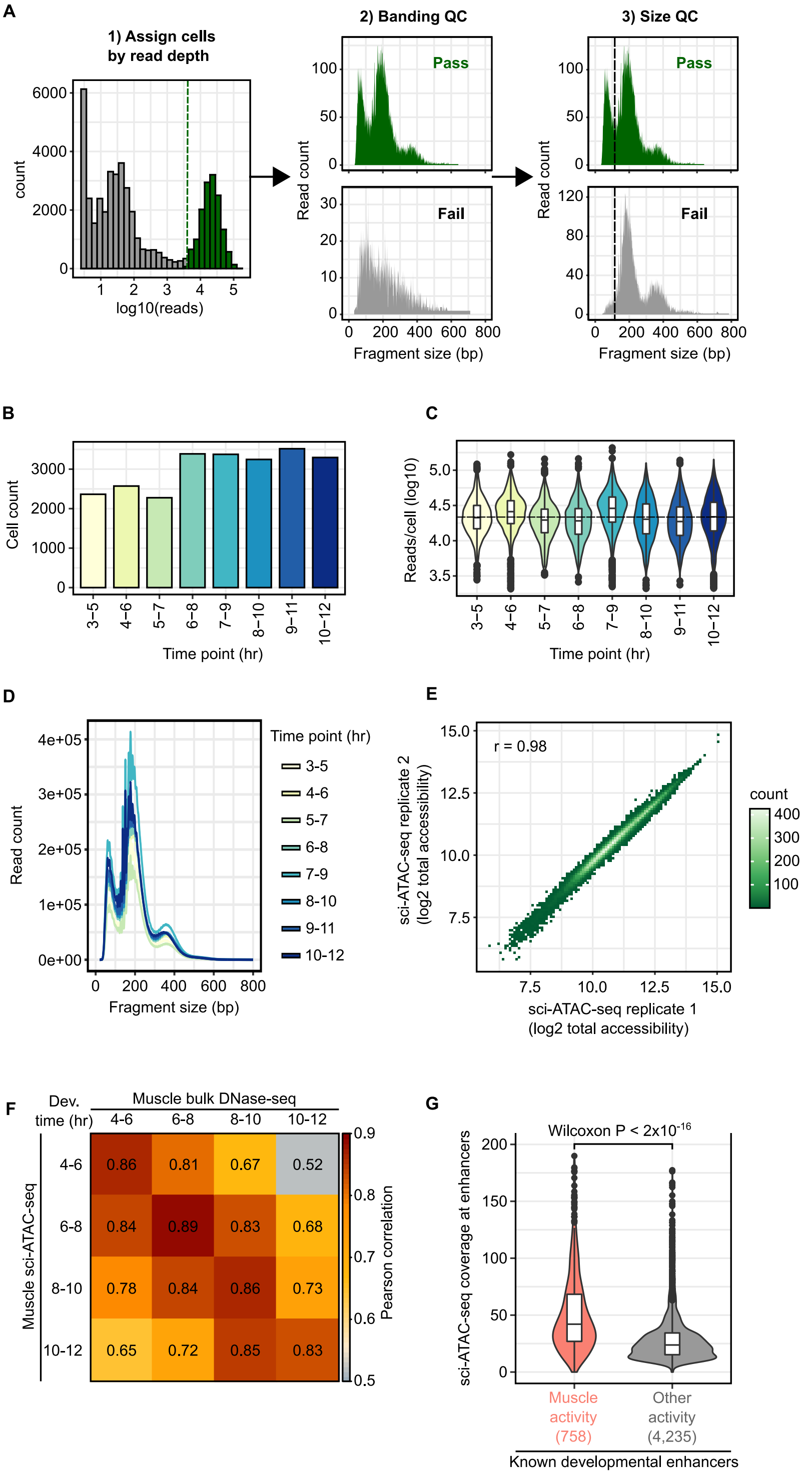

Figure S1 (related to Figure 1): sci-ATAC-seq during a dense time course of mesoderm development

(A) Quality control of sci-ATAC-seq libraries using a three-step filtering procedure. Only cells passing an initial read depth filter (step 1), with a clear nucleosome banding pattern (step 2) and fragment size distribution (step 3) were retained for downstream analysis. **(B)** Number of cells profiled per time point for the wild-type mesoderm/muscle time course. **(C)** Coverage (log10 unique reads) per cell per time point for the wild-type mesoderm/muscle time course. Dashed line indicates the median coverage per cell across the entire dataset (21,649 reads/cell). **(D)** Fragment size distribution per time point displays the expected nucleosome banding pattern for ATAC-seq libraries. **(E)** Correlation of sci-ATAC-seq replicates (batch 1 and 2) in accessibility across 42,076 merged ATAC-seq peaks called on each time point. r indicates the Pearson's correlation coefficient, showing little batch effect. **(F)** Correlation between bulk DNase-seq accessibility profiles from FAC sorted mesoderm/muscle cells (Reddington et al., 2020) and aggregated pseudobulk sci-ATAC-seq profiles for time-matched samples. **(G)** sci-ATAC-seq coverage at characterized mesoderm/muscle enhancers (red) and enhancers active in other embryonic tissues (non-muscle, grey).

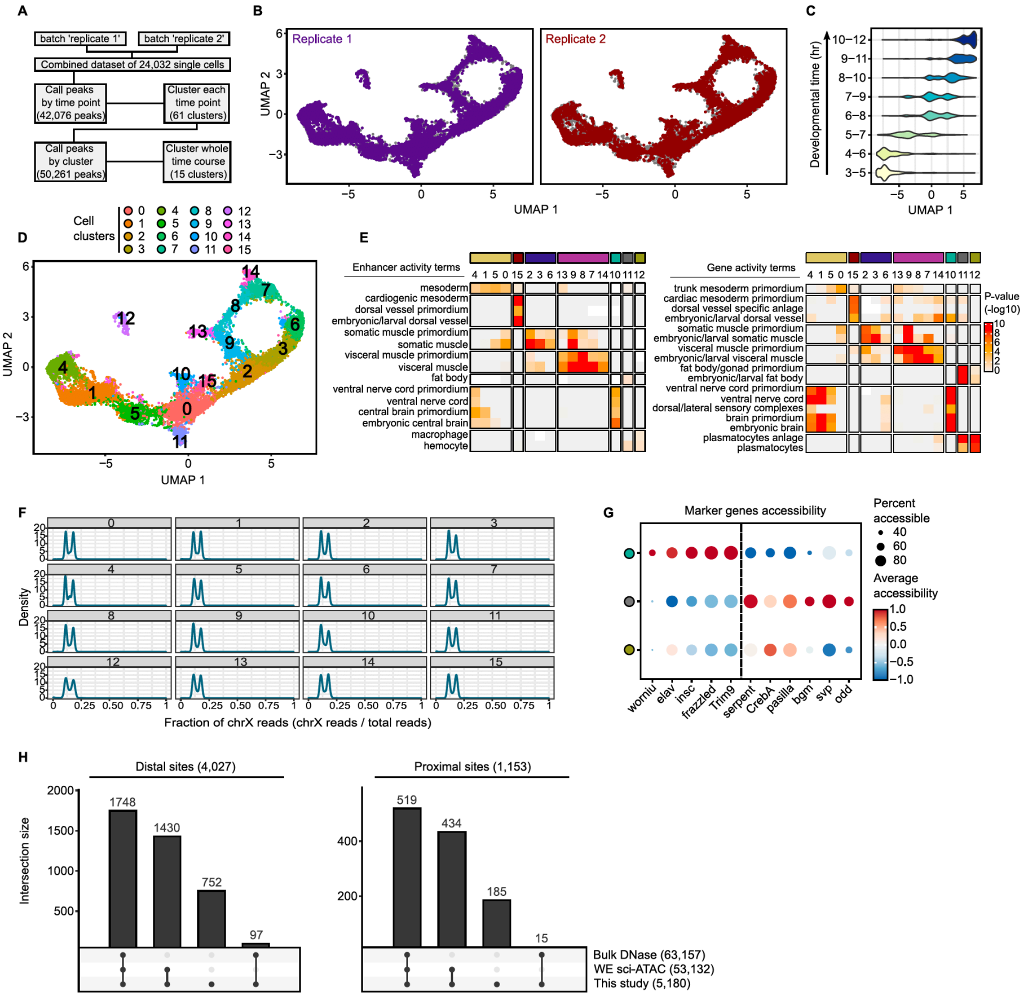

Figure S2 (related to Figures 1-2): Constructing, annotating and analyzing an atlas of chromatin accessibility during a time-course of mesoderm/muscle development

(A) Overview of data processing and clustering strategy. After cell filtering (Fig. S1A), the two sci-ATAC-seq batches were merged giving a combined set of 24,032 mesodermal (Mef2+) cells. To maximise sensitivity to detect peaks at each stage and cell type, peaks were first called separately for each timepoint (yielding a combined set of 42,076 peaks), which were used to cluster each time point individually (using Seurat v3.2.2), yielding 61 cell clusters. Accessibility peaks were identified again in each cell cluster, identifying 50,261 merged peaks used for clustering the whole wild-type time-course. **(B)** UMAP visualization of the whole mesoderm/muscle time course, highlighting cells belonging to each batch in purple (rep 1) or red (rep 2). Cells from each batch are evenly distributed throughout the time-course, suggesting little or no batch effect, keeping with the high correlation between batches (Fig. S1E). **(C)** Cell distribution over the first UMAP dimension per time point, showing the gradual progression along developmental time. **(D)** UMAP visualization of the full mesoderm/muscle time course with cells coloured by their respective Seurat defined cluster. **(E)** Enrichment for enhancers with tissue activity (left) and genes with tissue expression (right) terms in each cluster. Fisher's exact test P-value (-log10) displayed as a heatmap. Cell clusters are grouped by the inferred cell type annotations (top bars), colours correspond to Figure 1D. **(F)** As embryo collections are a mix of male and females, we assessed if there are sex-dependent effects. Each nucleus was molecularly sexed using the fraction of reads mapping to the X Chr, as female nuclei have roughly double the number of X-reads than males. Density plots show the fraction of reads from the X Chr (x-axis) for nuclei in each cluster (corresponding cluster number from (D) indicated on top). Each cluster has a bimodal distribution among nuclei in the ratio of X-Chr reads, indicating little or no sex-biases in each muscle cluster. **(G)** Marker genes accessibility for non-myogenic cell populations (top to bottom: neuro, fat body, hemocytes). The colour scale indicates gene average accessibility (Z-score), while dot size indicates the percentage of cells in which the gene is accessible. Annotation colours correspond to Figure 1D. **(H)** Upset plot showing the intersection between differentially accessible (DA) ATAC-seq peaks discovered here (5,180 peaks from Fig. 2D) compared to 63,157 peaks from tissue-specific bulk DNase-seq (Reddington et al., 2020) and 53,132 peaks from whole-embryo (WE) shot-gun sci-ATAC-seq (Cusanovich et al., 2018a). The DA sites are divided into distal (left, >500bp from a Transcriptional Start Site (TSS)) and TSS-proximal sites (right). An intersection is counted if at least 25% of a peak in this study overlaps a peak in another dataset.

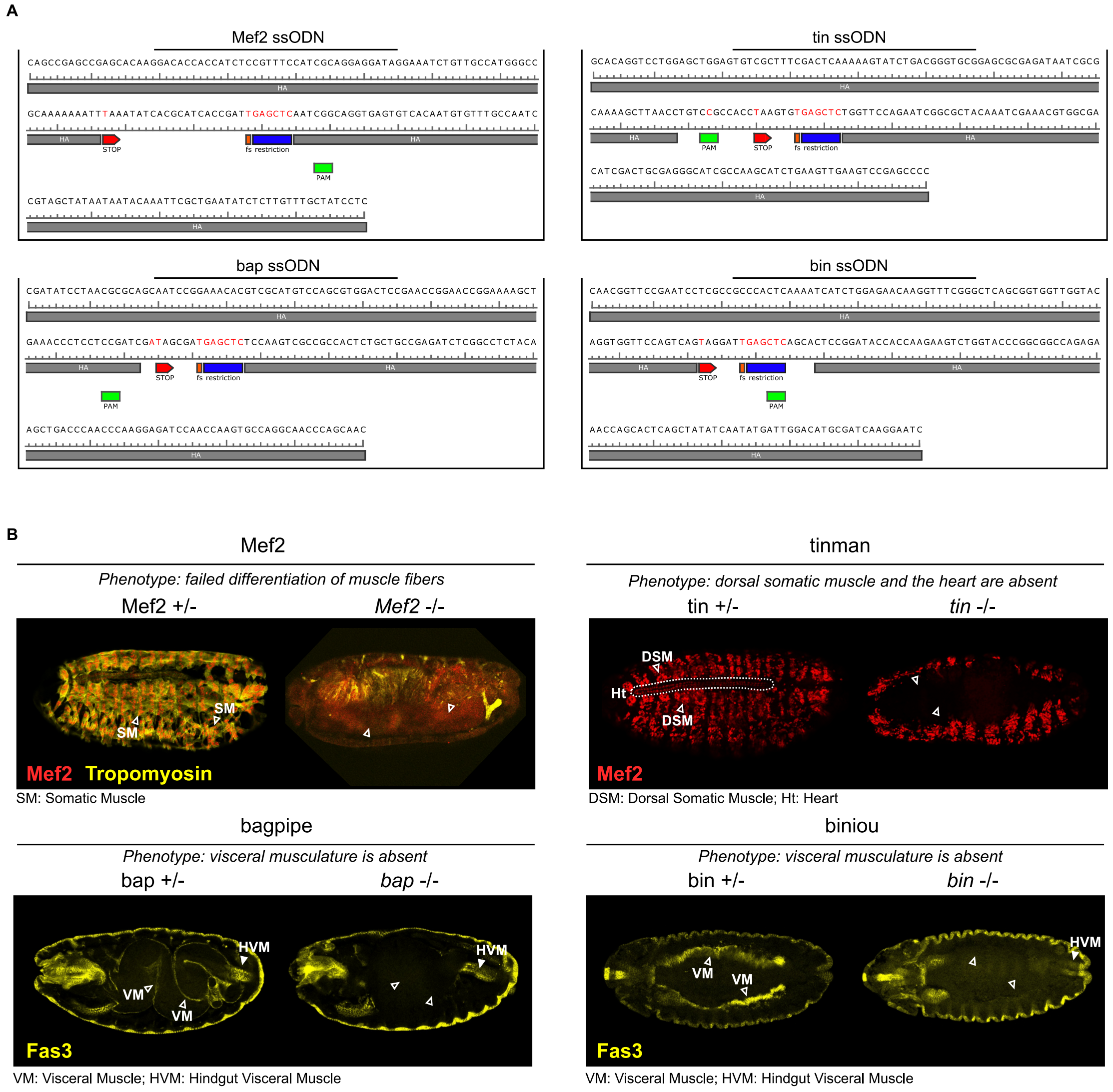

Figure S3 (related to Figures 4-5): Generation and validation of new loss-of-function mutants for four mesodermal transcription factors

(A) Design of the single stranded oligonucleotides (ssODNs) for the *Mef2*, *tin* (*tinman*), *bap* (*bagpipe*), and *bin* (*biniou*) loci, which were used as homology directed repair (HDR) templates following a Cas9 induced double strand break. Nucleotides that differ from the isogenic vasa-Cas9 locus are indicated in red. For *Mef2*, *tin* and bin, the introduced mutation recapitulates the characterised loss-of-function allele, but is now generated in a clean isogenic background common to all four mutants. This previously characterized single point nonsense mutation (indicated by the red "STOP" box), in addition to a restriction site for SacI (GAGCTC; blue box) and a thymine nucleotide causing a nonsense frame-shift mutation (orange box) were introduced in each locus. Additional single point mutations were introduced in either the PAM site or the gRNA seed to prevent re-cutting by Cas9. **(B)** All four CRISPR generated new alleles recapitulate the characterised loss-of-function phenotypes for each gene. Upper left panels: Double immunostaining of Mef2 (red) and Tropomyosin (yellow) in heterozygous (+/-; identified by LacZ expression on the balancer chromosome) and homozygous (-/-) *Mef2* mutant stage 16 embryos. While the *Mef2* +/- embryo has stereotypically patterned somatic muscle (SM), the *Mef2* -/- embryo has an absence of Mef2 protein and little or no differentiated somatic muscle. Upper right panels: Immunostaining of Mef2 (red) in *tin* heterozygous (+/-) and homozygous (-/-) mutant stage 16 embryos. The dorsal somatic muscle (DSM) and heart muscle (Ht) indicated in *tin* +/- embryos are missing in the *tin* -/- embryo, in addition to the visceral muscle (not shown). Lower panels: Immunostaining of Fas3 (yellow) as a marker for visceral muscle (VM) in *bap* (left) and *bin* (right) heterozygous (+/-) and homozygous (-/-) mutant embryos. Note the complete absence of circular trunk visceral muscle in both mutants, while the hindgut VM (HVM) is unaffected. All embryos are orientated anterior to the left, and dorsal up.

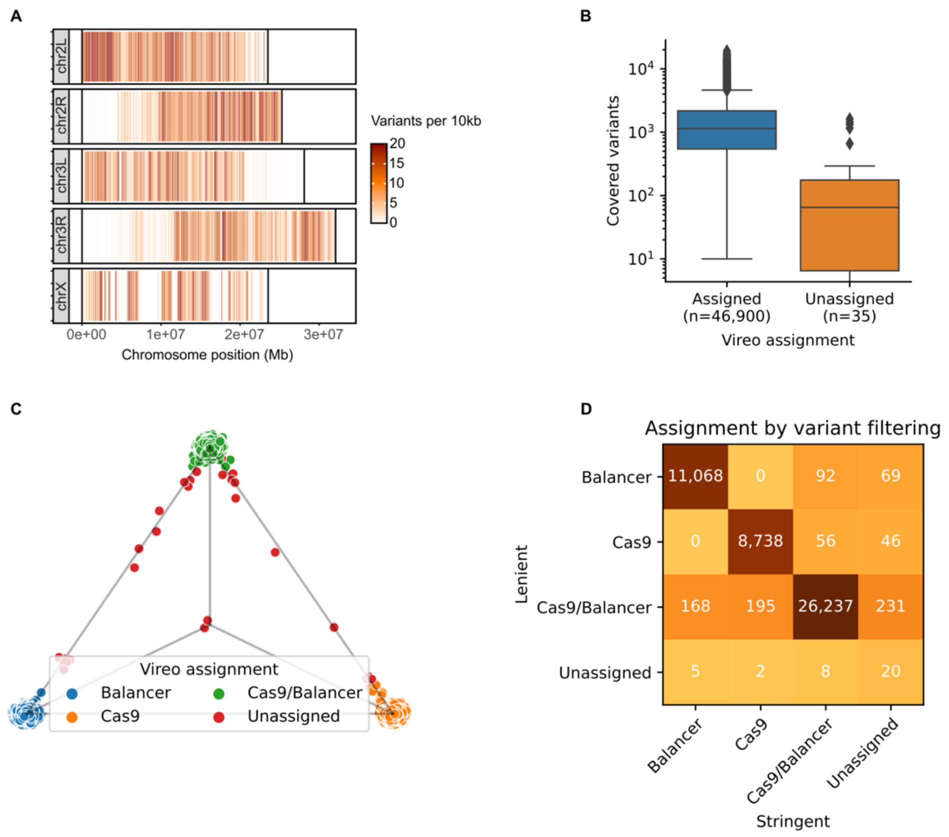

Figure S4 (related to Figures 4-5): Digital nuclear genotyping

(A) The density of genetic variant between the Cas9 isogenic strain used to make the loss-of-function mutants (sequenced in this study) and the balancer chromosome (sequenced in our previous study (Ghavi-Helm et al, 2019) across chromosomes 2, 3 and X. **(B)** Box plots showing the total number of SNP variants per nuclei with a genotype assigned (blue) or unassigned (orange) by Vireo (Huang et al., 2019). **(C)** Triangle plot showing assignment probabilities for each genotype, which are represented as points in the triangle. The center of the triangle corresponds to the vector (1/3, 1/3, 1/3), while the vertices represent a probability of 1 for Balancer (bottom left), Cas9 (bottom right) and Cas9/Balancer (top), respectively. Posterior probabilities are focused near the vertices of the triangle, indicating low posterior uncertainty. **(D)** Confusion matrix for the genotype assignment using variants called with a stringent or a lenient filter, demonstrating that the model is robust to different reference annotations.

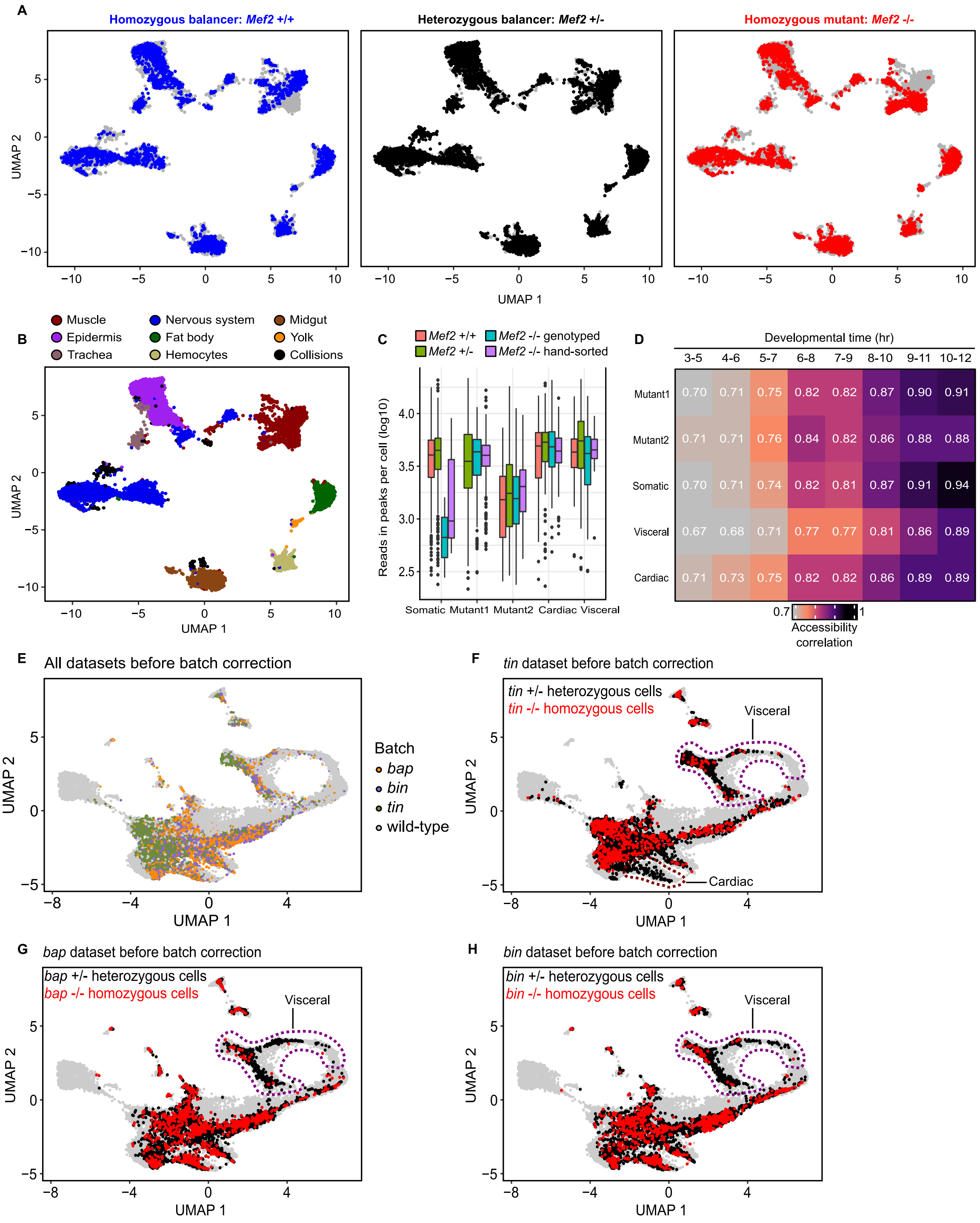

Figure S5 (related to Figures 4-5): Clustering, annotation and analysis of *Mef2*, *tin*, *bap*, *bin* mutants
(A) UMAP visualization of whole-embryo cell clustering from the *Mef2* sample; homozygous balancer/balancer (left, blue), heterozygous Mef2^{lof}/balancer (+/-, middle, black), homozygous Mef2^{lof} (-/-, right, red) cells are highlighted. Cell clustering is not obviously impacted by genotype, indicating little batch effect and therefore no need for correction. **(B)** Same as (A), cells coloured by inferred cell type, revealing 8 populations, including muscle cells (red). **(C)** Reads in peaks per cell (log10 scale) per cell cluster from Fig. 4d, split by genotype. Mutant 2 cluster has lower read counts/cell. **(D)** Pearson correlation of open chromatin for clusters in Fig. 4d in time point aggregate samples from the wild-type time course. Mutant 1 cells have the highest correlation to cells at 10-12hr, matching the specific time point of their sampling (10-12 hr). **(E)** UMAP visualization before Harmony batch correction of wild-type sci-ATAC-seq time course re-clustered with single cell datasets from *tinman* (*tin*), *bagpipe* (*bap*) and *biniou* (*bin*). All cells from each dataset (batch) are highlighted (*bap* - orange, *bin* - purple, *tin* - green, wild-type - grey). **(F-H)** Same as (E), but for each dataset (batch) the heterozygous (+/-; black) and homozygous mutant (-\-; red) cells are highlighted. The cardiac and visceral muscle populations are highlighted with dashed lines.

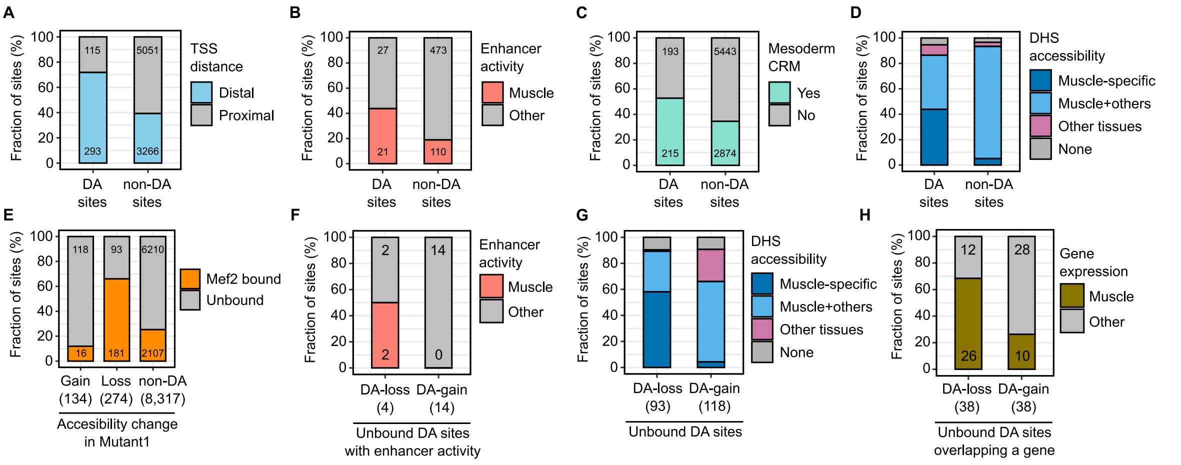

Figure S6 (related to Figure 6): Characterization of regulatory changes in *Mef2* mutants

(A) Fraction of differentially accessible (DA) or non-DA sites in Mutant 1 cells that are gene distal (light blue) or gene proximal (grey). Gene distal is defined as >500bp from an annotated transcriptional start site. **(B)** Fraction of *Mef2* -/- DA or non-DA sites that overlap characterized embryonic enhancers with demonstrated activity in muscle (red) or other tissues (grey). **(C)** Fraction of *Mef2* -/- DA or non-DA sites overlapping mesoderm *cis*-regulatory modules (CRMs), defined by ChIP against 5 mesoderm/muscle transcription factors (Zinzen et al., 2009). **(D)** Fraction of DA or non-DA sites overlapping DNase I Hypersensitive Sites (DHSs) in muscle only (Muscle-specific), in muscle and other tissues (Muscle+others), in non-muscle tissues (Other tissues) or that do not overlap a DHS (None). DHS data from Reddington et al, 2020. **(E)** Fraction of Mef2-bound (orange) and unbound (grey) sites, split by chromatin accessibility change in Mutant1 cells, either gain or loss of accessibility, or unchanged (non-DA) sites. **(F)** Fraction of Mef2-unbound DA sites that lose (DA-loss) or gain (DA-gain) accessibility in Mutant1 overlapping characterised enhancers active in muscle (red) or other tissues (grey). **(G)** Fraction of Mef2-unbound DA sites that lose (DA-loss) or gain (DA-gain) accessibility in Mutant1 that overlap DHS in muscle only (Muscle-specific), in muscle and other tissues (Muscle+others), in non-muscle tissues (Other tissues) or that do not overlap a DHS (None). DHS data from Reddington et al, 2020. **(H)** Fraction of Mef2-unbound DA sites that lose (DA-loss) or gain (DA-gain) accessibility in Mutant1 overlapping genes with characterized expression in muscle (olive) or non-muscle tissues (other, grey). Gene expression data from the BDGP database.
